# A genetically targeted sensor reveals spatial and temporal dynamics of acrosomal calcium and sperm acrosome exocytosis

**DOI:** 10.1016/j.jbc.2022.101868

**Published:** 2022-03-27

**Authors:** Roy Cohen, Chinatsu Mukai, Jacquelyn L. Nelson, Shoshana S. Zenilman, Danielle M. Sosnicki, Alexander J. Travis

**Affiliations:** 1 Baker Institute for Animal Health, College of Veterinary Medicine, Cornell University, Ithaca, New York, USA; 2 Department of Public and Ecosystem Health, College of Veterinary Medicine, Cornell University, Ithaca, New York, USA

**Keywords:** sperm, microscopy, fertilization, mouse, acrosome exocytosis, ACR, acrosomal Ca2+ rise, AcroSensE, Acrosome-targeted Sensor for Exocytosis, AE, acrosome exocytosis, APM, acrosomal plasma membrane, CD, 2-hydroxypropyl-β-cyclodextrin, FP, fusion pore, MF, membrane fusion, OAM, outer acrosomal membrane, PSF, prespike foot, RNE, redundant nuclear envelope, SERCA, sarcoplasmic/endoplasmic reticulum Ca^2+^-ATPase, SPCA, secretory pathway Ca^2+^ ATPase, STIM, stromal interaction molecule, ZP, zona pellucida

## Abstract

Secretion of the acrosome, a single vesicle located rostrally in the head of a mammalian sperm, through a process known as “acrosome exocytosis” (AE), is essential for fertilization. However, the mechanisms leading to and regulating this complex process are controversial. In particular, poor understanding of Ca2+ dynamics between sperm subcellular compartments and regulation of membrane fusion mechanisms have led to competing models of AE. Here, we developed a transgenic mouse expressing an Acrosome-targeted Sensor for Exocytosis (AcroSensE) to investigate the spatial and temporal Ca2+ dynamics in AE in live sperm. AcroSensE combines a genetically encoded Ca2+ indicator (GCaMP) fused with an mCherry indicator to spatiotemporally resolve acrosomal Ca2+ rise (ACR) and membrane fusion events, enabling real-time study of AE. We found that ACR is dependent on extracellular Ca2+ and that ACR precedes AE. In addition, we show that there are intermediate steps in ACR and that AE correlates better with the ACR rate rather than absolute Ca2+ amount. Finally, we demonstrate that ACR and membrane fusion progression kinetics and spatial patterns differ with different stimuli and that sites of initiation of ACR and sites of membrane fusion do not always correspond. These findings support a model involving functionally redundant pathways that enable a highly regulated, multistep AE in heterogeneous sperm populations, unlike the previously proposed “acrosome reaction” model.

The “acrosome reaction” model involves receptors on the sperm membrane being triggered by physical contact with ligands on the zona pellucida (ZP), resulting in exocytosis of the acrosome, the sperm’s single exocytotic vesicle. This long-dominant model defines two states or conditions for sperm—acrosome-intact or acrosome-reacted. In this model, it is thought that creating pores between the plasma membrane and outer acrosomal membrane (OAM) would result in loss of acrosomal contents in a manner analogous to popping a water balloon. Almost 2 decades of data challenge this thinking, suggesting a more complex, multistep model of “acrosome exocytosis” (AE). However, the acrosome reaction model has proven remarkably persistent, due both to its simplicity and lack of precise understanding of the steps involved in AE. The multistep AE model is based on numerous lines of evidence from different laboratories, including that putative receptors for the ZP are found in the acrosomal matrix (1) and then exposed on the sperm surface (2); that AE occurs during transit through the cumulus cells prior to the sperm reaching the ZP (3); that contact with the ZP is insufficient to induce AE in sperm expressing GFP in their acrosome (AcrGFP sperm), even several hours after binding (4); that the majority of sperm have undergone AE even before reaching the cumulus mass (5); and that only ∼5% of the sperm traversing the ampulla are acrosome-intact (6). However, there are contradictions even within these reports, and the field remains highly controversial (7) and in need of new tools to understand the regulation and process of exocytosis.

A critical knowledge gap in understanding AE results from challenges in studying sperm Ca^2+^ dynamics. There are multiple factors underlying this, including (A) sperm are transcriptionally and translationally inactive with no culture systems available to produce/manipulate mature sperm; (B) mature sperm are motile and difficult to image; (C) across mammalian species, only a subset of sperm are actually capable of fertilizing (8, 9, 10, 11, 12, 13, 14) and these have different Ca^2+^ dynamics (15); (D) sperm only acquire the ability to fertilize after undergoing functional maturation in a complex process known as “capacitation” involving multiple changes in membrane lipids such as removal of sterols, polarization state, and ion flux (16, 17, 18); (E) sperm membranes are remarkably temperature sensitive with phase transitions occurring between physiological and room temperatures (19, 20); and (F) there is scant cytoplasmic space between the plasma and OAMs, making it difficult to discern differences in Ca^2+^ dynamics within cytoplasmic *versus* acrosomal compartments without a compartmentalized marker.

We previously described how removal of cholesterol and focal enrichments of G_M1_ play a regulatory role in enabling AE through at least 2 complementary pathways in the plasma membrane overlying the acrosome (acrosomal plasma membrane [APM]). First, sterol efflux enables proteolytic activation of phospholipase B, which cleaves both tails of a phospholipid molecule (21, 22), changing local membrane curvature to support point fusion formation. Second, sterol efflux enables G_M1_ regulation of the Ca_V_2.3 voltage-gated Ca^2+^ channel, giving rise to Ca^2+^ transients that facilitate AE in response to subsequent Ca^2+^ waves in the sperm’s head (23). Along with these roles, sterol efflux also exerts broad regulatory functions, such as facilitating intracellular alkalinization through activation of the sperm proton channel Hv1 in human sperm (24) or sperm-specific sodium hydrogen exchanger in murine sperm (25) and plasma membrane hyperpolarization through activation of the SLO3 potassium channel (26). Both alkalinization and hyperpolarization are critical steps for capacitation and AE to occur (27, 28).

As a complement to these membrane lipid–regulated pathways, progesterone (P4) is also a critical regulator of sperm function. Released by cumulus cells and encountered by sperm in the vicinity of the egg, P4 has been reported to facilitate multiple events in sperm including stimulation of capacitation (29), hyperactivation and flagellar bending (30), chemotaxis (31, 32), and AE (33, 34, 35). Notably, P4 induces Ca^2+^ entry into sperm (36); these Ca^2+^ elevations are mediated by the CatSper channel (37, 38). Patch clamping data have sometimes been interpreted to suggest that CatSper is the only active Ca^2+^ channel in sperm, as opposed to previous models in which multiple channels contribute to Ca^2+^ transients and waves that culminate in release from internal stores and activation of store-operated channels.

To investigate some of the complexities of AE and Ca^2+^ dynamics in sperm, we generated a novel mouse model with a genetically encoded Ca^2+^-sensor (GCaMP3) in fusion with mCherry that we targeted to the acrosome. This positions it both to reflect Ca^2+^ concentrations in that compartment and reflect the status of the acrosomal contents, in live sperm, in real time, under physiological conditions. As a component of the matrix, loss of the photostable and pH-insensitive mCherry fluorescence from the sperm would reflect membrane fusion (MF) and AE akin to the acrosome-targeted GFP mouse line (2, 39, 40). We hypothesized that this combination of features could also reveal fusion pore (FP) formation as indicated by Ca^2+^ influx into the acrosome lumen, a point of contention between the models.

We selected a GCaMP variant for our transgenic mouse line based on reported Ca^2+^ concentrations in the acrosome of between 256 μM and 1.02 mM (41). This led us to choose a lower affinity variant GCaMP (GCaMP3/GCaMPer) originally developed to monitor Ca^2+^ in the endoplasmic reticulum. GCaMP3 has a Kd of approximately 400 μM, with good dynamic range for Ca^2+^ concentrations between 10^−4^ and 10^−3^ M (42). In addition to the GCaMP component, we also added the bright monomeric mCherry as a non–Ca^2+^-sensitive fluorescent protein to enable visualization of the acrosome while the GCaMP3 signal is dim (*e.g.*, prior to Ca^2+^ binding). We targeted the GCaMP–mCherry fusion protein to the acrosome using the acrosin promotor and signaling peptide (39).

Here, we characterize this novel model (Acrosome-targeted Sensor for Exocytosis [AcroSensE]) and then use it experimentally to inform our understanding of AE and sperm Ca^2+^ dynamics. For these initial studies, we chose stimuli that sperm would encounter in the vicinity of the egg (P4) or that would mimic sterol acceptors found in the female tract in a reductionist, controlled way (*i.e.*, 2-hydroxypropyl-β-cyclodextrin [CD] as a chemically defined mediator of sterol efflux *versus* oviductal albumin or high-density lipoproteins), or stimuli that exist on sperm but are released from inhibition provided by the binding of seminal plasma proteins (G_M1_). Data generated from these AcroSensE mice support a complex, multistep AE model and are inconsistent with the traditional acrosome reaction model, helping resolve the existing controversy and providing new insights into functionally redundant mechanisms regulating sperm AE.

## Results

### Production and validation of the AcroSensE mouse model

**Figure 1:**
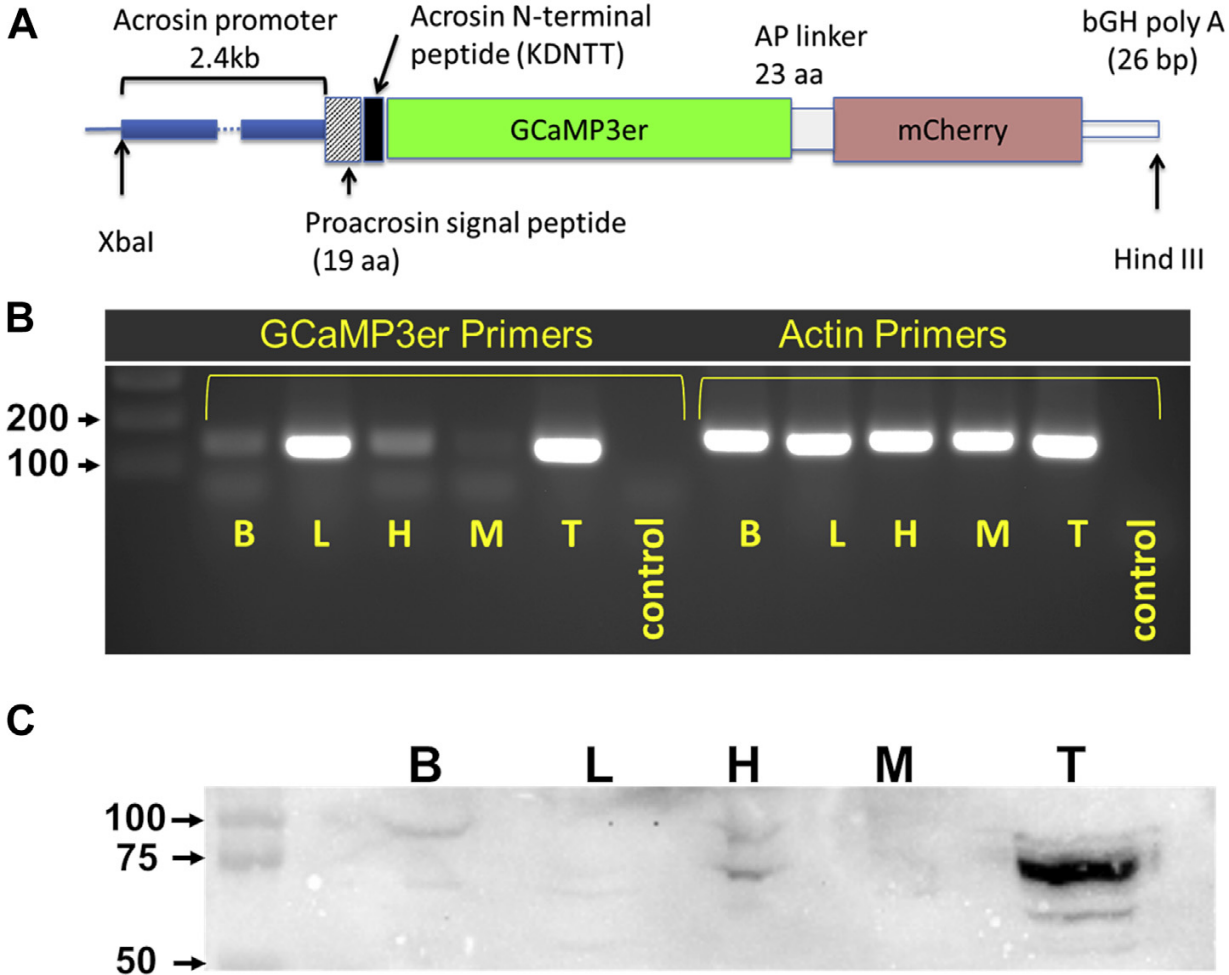
**Design and validation of the mouse line expressing Acr-GCaMP3er-mCherry (Acrosome-targeted Sensor for Exocytosis [AcroSensE]).***A*, schematic of the construct. *B*, validation of Acr-GCaMP3-mCherry expression in the model using RT-PCR in various tissues (brain (B), liver (L), heart (H), muscle (M), testis (T), and negative control). Primer sets were designed to amplify 149 base pairs between the proacrosin signal peptide and the GFP portion of the GCaMP3 (f’:catggtcctgctggagttcgtg, r’:ctggtcgagctggacgggcgacg). Actin was used as a positive control. *C*, immunoblot analysis of protein expression using anti-GFP confirmed high levels of expression of Acr-GCaMP3-mCherry in the testis at the predicted molecular weight of 75 kDa.

To generate the AcroSensE plasmid, we replaced the GFP sequence in the pAcr3-EGFP backbone plasmid (39) with a cassette comprising the low-affinity GCaMP3 (42) and mCherry in fusion (Fig. 1*A*). The mCherry and GCaMP3 were coupled by a 23-aa linker (flexible alanine–proline repeat linker (43). Transgenic founders were obtained by injecting the linearized AcroSensE plasmid into the pronucleus of 100+ fertilized embryos obtained by mating C57BL6/J and CBA F1 hybrids (Stem Cell and Transgenic Core Facility). Transgenic founders were backcrossed to C57BL/6 mice. Insertion of the target construct in the transgenic founders was verified by genomic PCR, using primers targeting the GCaMP3 region of the AcroSensE construct. To validate expression of the transgene, we first determined the expression levels of AcroSensE in various tissues. Figure 1*B* shows the results of RT-PCR analysis of AcroSensE demonstrating the highest transcript levels in the liver and testes. However, immunoblots of protein extracts using an antibody against the GFP backbone of the GCaMP3 (1 ng/μl, Abcam, Cambridge, MA) demonstrated that the AcroSensE protein was expressed predominantly in testes (Fig. 1*C*). Moreover, we were not able to detect expression of the AcroSensE construct at the protein level in any tissue but sperm using fluorescence microscopy (detected *via* the mCherry fluorescence; data not shown).

### Resolution of intermediate MF steps during AE

Sperm from AcroSensE mice were collected as previously described (23). Following incubation for 30 to 60 min at 37 °C in the presence or absence of 3 mM CD and 10 mM bicarbonate as indicated, sperm were placed on a polylysine-coated coverslide dish (MatTek Corp) and imaged on a Zeiss LSM 510, with a controlled delivery system for local administration of ionophore A23187, G_M1_, CD, or P4. Preincubation of populations of sperm under capacitating conditions was performed with some stimuli (CD with/without bicarbonate) to mimic the physiological timeline when sterol efflux would occur relative to the effects of those other molecules (*e.g.*, G_M1_ and P4). To stimulate exocytosis in single cells, a short (10 s) puff of stimulant was applied near an individual sperm *via* micropipette while recording the changes in GCaMP3 and mCherry fluorescence intensities at image rates of up to 5 frames per second for each wavelength. Concentrations are indicated for the solution in the micropipette, as specified in the Experimental procedures section.

To understand baseline effects and aid interpretation, we quantified the percentage of w.t. B6 sperm undergoing AE as a result of initial incubation under noncapacitating and capacitating conditions (Fig. S1). Using Coomassie staining, we found that 21.7% of the cells underwent AE in response to a 30-min incubation (at 37 °C) with 3 mM CD or 24.6% of cells with 25 μM G_M1_ (data from 3 mice each, counting at least 180 cells in each condition). Under noncapacitating control conditions (no CD or G_M1_), 10.7% of sperm underwent AE. After 90 min, 36.3% of sperm underwent AE in response to G_M1_, 45.2% in response to CD, and 47.1% in response to both CD and G_M1_. At this time point, the value for noncapacitating conditions also increased to 25%. These percentages are similar to our prior observations (23). Because the sperm that responded to the preincubation by undergoing MF and AE would have lost both their GCaMP3 and mCherry signals prior to encountering the other stimuli in our experimental approach, they would not be observed and quantified in the live imaging experiments. That is, any sperm undergoing spontaneous AE or AE in response to preincubation did not contribute to the subpopulations showing acrosomal Ca^2+^ rise (ACR) and MF upon stimulation with various reagents mentioned in the following part of the article.

After preincubation for 30 min, the AcroSensE sperm were then imaged. In each trial, sperm from multiple mice were pooled, and typically only 1 to 2 of the experimental conditions described here were tested per trial to ensure that length of preincubation would not be a confounding factor in interpreting results from different conditions. Under these experimental conditions, we observed several steps in the process leading to AE, consistent with ACR, and full MF leading to loss of the mCherry fluorescence and therefore full AE. In some cells, we saw traces similar to prespike foot (PSF) events as frequently observed when using amperometry to study exocytosis; however, these were on a much slower time course than when observed in other cells using amperometry. Although temporally these PSF-like events were the earliest events we observed using AcroSensE fluorescence, they were observed infrequently and only under specific circumstances. Therefore, we will first describe the more common ACR and MF responses to the different treatment conditions.

**Figure 2:**
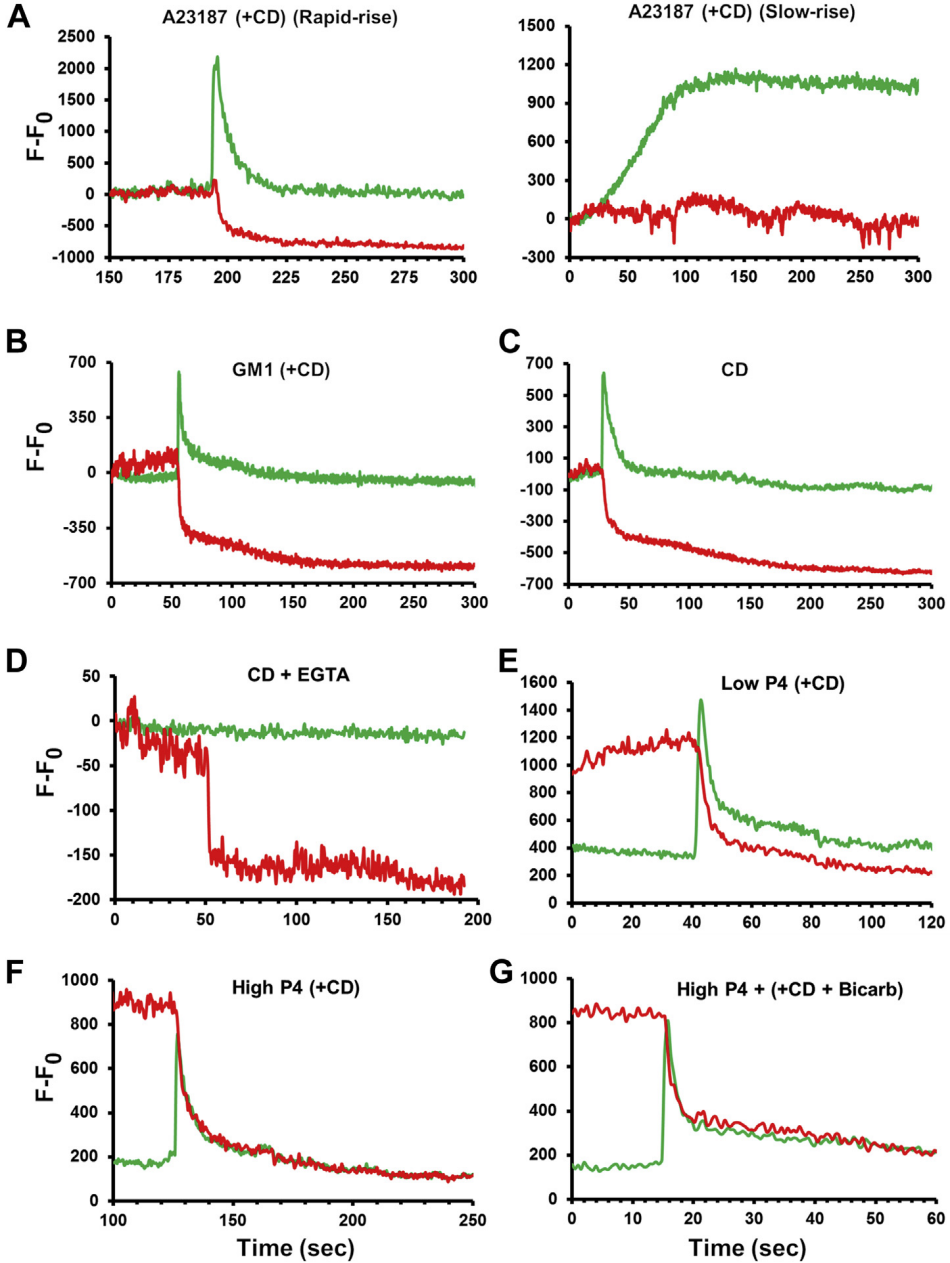
**Representative traces of changes in fluorescence reflecting different Ca**^**2+**^**and membrane fusion dynamics under various conditions.** Traces are provided as the fluorescence signal (F) after subtracting the fluorescence intensity at time point zero (F_0_; F-F_0_). *A*–*D*, representative traces of changes in acrosomal Ca^2+^ rise (ACR) resulting from increase in GCaMP3 fluorescence (*green*) and membrane fusion (MF), resulting from loss of mCherry fluorescence (*red*) following the addition of (*A*) ionophore A23187 (50 μM puff; estimated final concentration ∼10 μM) where we observed 2 distinct responses: rapid rise (*left panel*) and slow rise (*right panel*); (*B*) G_M1_ (125 μM puff; final concentration ∼25 μM); (*C*) 2-hydroxypropyl β-cyclodextrin (CD; 20mΜ; final concentration ∼4 mM); (*D*) CD (4 mM) + EGTA (8 mM). Note that this specific trace (*D*) was chosen to demonstrate that the AcroSensE construct was functional, indicated by the loss of the mCherry signal, although no rise in GCaMP3 fluorescence was observed. The majority of cells under this condition did not undergo MF/AE. *E*–*G*, representative traces provided for (*E*) Low P4 (15 μM puff; final concentration ∼3 μM); (*F*) High P4 (300 μM puff; final concentration ∼60 μM), and (*G*) High P4 + bicarb + CD (final concentration of P4 ∼60 μM; 10 mM bicarb was only added during the incubation period). AcroSensE, Acrosome-targeted Sensor for Exocytosis.

**Figure 3:**
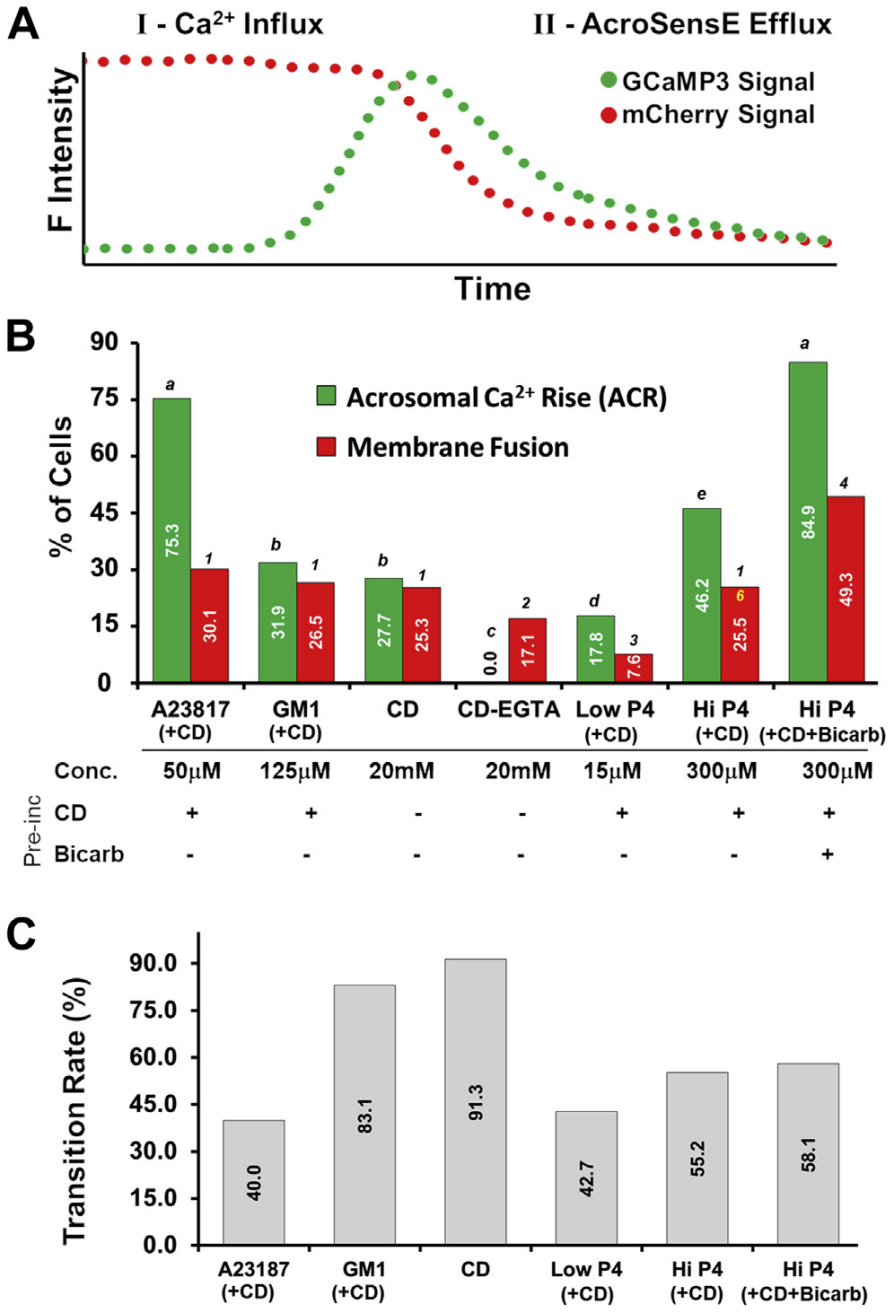
**Using AcroSensE to resolve acrosomal Ca**^**2+**^**rise (ACR) and membrane fusion (MF) during acrosome exocytosis.***A*, illustration of the changes over time in the CGaMP3 (*green wavelength*) and mCherry (*red wavelength*) fluorescence intensity signals. Increase of the GCaMP3 signal is a result of Ca^2+^ binding, while the loss of the mCherry signal is attributed to the loss of the AcroSensE protein out of the acrosome to the extracellular space. Note that the x-axis is expanded relative to Figure 2. *B*, summary of the percentage of cells demonstrating ACR (*green bars*) and MF (loss of mCherry signal, *red bars*) following A23187 (n = 93), G_M1_ (n= 113), CD (n= 166), CD +EGTA (n= 164), *Low* P4 (n = 115), *High* P4 (n = 208), and *High* P4 + bicarb (n = 73). All concentrations identical to those in Figure 2. “+” or “-” indicate whether sperm were preincubated with 3 mM CD and/or 10 mM bicarbonate, respectively. Significant differences between conditions as measured by χ^2^ (*p* < 0.01) are indicated by letters (for ACR) or numbers (for MF). In this and all subsequent figures, the appearance of the same letter or number shows no difference when comparing the values for those conditions. *C*, summary of the transition rates (% of cells) of sperm exhibiting transition from Ca^2+^ rise to MF, as calculated from (*B*). AcroSensE, Acrosome-targeted Sensor for Exocytosis; CD, 2-hydroxypropyl β-cyclodextrin.

Figure 2 provides traces demonstrating either representative changes in the GCaMP3 and mCherry fluorescence intensities following various stimulations or changes shown in specific subpopulations of sperm deserving special mention (Fig. 2, *A* and *D*; note that population data are summarized in Fig. 3, *B* and *C*). Following the addition of ionophore A23187, one subpopulation of sperm demonstrated rapid ACR which was typically quickly followed by MF as evidenced by loss of mCherry signal (Fig. 2*A*, left panel; 40% of responding sperm). In contrast, the remaining 60% of sperm responding to A23187 showed a slow, gradual ACR that did not result in AE within the time frame of image acquisition (until AE occurred or up to 8 min, Fig. 2*A* right panel).

Stimuli resulting in alterations in membrane lipids (*i.e.*, sterol efflux mediated by addition of CD and sterol efflux coupled with exogenous G_M1,_ Fig. 2, *B* and *C*) typically resulted in a lower proportion of cells responding, but more uniformity in having a rapid ACR, followed quickly by MF in most of the responding sperm. The notable exception was the case of CD stimulation in the absence of extracellular Ca^2+^ (CD + EGTA), in which no ACR was observed. Interestingly, although the EGTA sufficiently reduced Ca^2+^ in this condition such that there was no detectable ACR (Figs. 2*D*), 17.1% of sperm still underwent exocytosis (shown here in Fig. 2*D* by loss of mCherry fluorescence; traces representing the majority of sperm that did not undergo MF and AE are not shown; see also Fig. 3*B* and discussion in the later part of the article).

Both low and high P4 conditions (with and without bicarbonate) induced ACR in varying percentages of sperm, with several differences seen in patterns of Ca^2+^ rise (more on spatial and temporal dynamics in figures). Excepting the CD+EGTA treatment, under all other conditions in which MF and AE were observed, a rise in GCaMP3 signal preceded the loss of mCherry fluorescence. These results combine to suggest that ACR resulted from extracellular Ca^2+^ entry into the acrosome and occurred prior to exocytosis of the acrosomal contents. This temporal pattern is illustrated in Figure 3*A* and further discussed.

Figure 3*A* provides a schematic representation of the typical observations for the AcroSensE sperm, in which time (X axis) is expanded relative to Figure 2, to highlight that the response to stimulation initiates with an increase in the GCaMP3 fluorescence intensity (reflecting ACR), followed by the loss of the mCherry and then loss of the GCaMP3 fluorescent signals. Figure 3, *B* and *C* summarize population data of the ACR and MF responses of cells to the various stimulations. In response to the addition of ionophore (A23187) following preincubation with 3 mM CD, 75.3% of the cells exhibited ACR and 30.1% exhibited MF (Fig. 3*B*). As described earlier in the study, 60% of cells responding to A23187 with ACR did not undergo MF, out of which 10.6% had fast ACR and 82% of cells had slow ACR (89.4% of sperm with fast ACR and 18% with slow ACR did progress into full MF).

In contrast, adding exogenous G_M1_ (following preincubation with CD) to the sperm’s plasma membrane, or induction of sterol efflux by addition of CD alone (with none added during preincubation), resulted in a lower percentage of cells responding with ACR than that responding to A23187 (31.9% and 27.7%, respectively, Fig. 3*B*). However, the vast majority of these responding cells also exhibited MF/AE (Fig. 3, *B* and *C*; G_M1_ + CD = 83.1% of ACR-positive cells underwent MF/AE; CD = 91.3% of ACR-positive cells underwent MF/AE). Note that the value of 25.3% of sperm undergoing MF in response to single-cell application of CD without prior exposure during preincubation is very close to what we saw when preincubating populations of sperm with CD as noted earlier, giving us confidence that exposure to this mediator of sterol efflux under these conditions of single-cell application had similar effectiveness regardless of the method of exposure. Recall that because preincubation with CD occurred first, and then the G_M1_ was added, those cells responding to G_M1_ represent a subpopulation that had not already responded to CD-mediated sterol efflux alone within the preincubation time frame. The combined percentage of sperm exhibiting MF in response to modulation of these two membrane lipids was therefore approximately 45% (calculated as the sum of 24.6% responding to CD alone and 26.5% of the remaining 75%, which is almost identical to prior results for exposure to both stimuli (23)). This reinforces the complementary nature of the multiple membrane lipid–dependent pathways that promote AE, but the combined results suggest that the total pool of AE-capable sperm is roughly 45%–50% regardless of the membrane lipid stimulus.

Comparison of responses under the other conditions revealed very different percentages of sperm experiencing ACR (Fig. 3*B*) but roughly comparable transition rates to that seen with A23187 (A23187: 40.0%, Low P4: 42.7%, Hi P4: 55.2%, Hi P4 + Bicarb: 58.1%; Fig. 3*C*). However, keep in mind that of sperm that responded to A23187 quickly, 89.4% underwent MF/AE, more consistent with CD alone and G_M1_ + CD.

### Spatial characterization of the steps leading to AE

**Figure 4:**
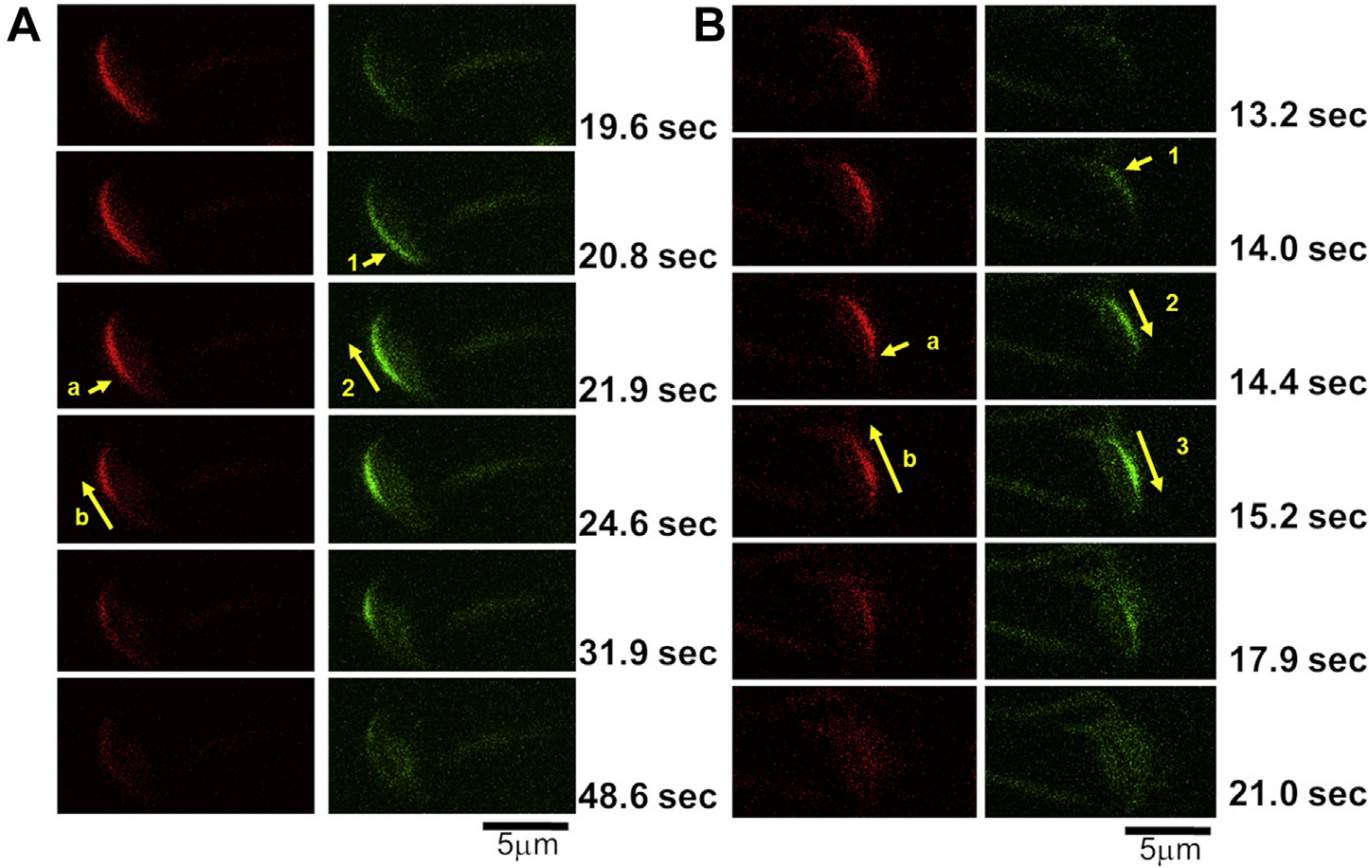
**Representative images of AcroSensE sperm undergoing different spatial patterns of acrosomal Ca**^**2+**^**rise (ACR), membrane fusion (MF), and acrosomal exocytosis (AE)**. *A*, visualization of *bottom-to-top* spatial progression of ACR and MF following addition of *high* P4 (+bicarb + CD) to AcroSensE sperm. The initial basal signal of the GCaMP3 is *low*, while maximal mCherry intensity is detected throughout the apical acrosome. Following stimulation, an increase in the GCaMP3 signal is detected near the *bottom* of the plasma membrane overlying the acrosome (*arrow* 1). The *green signal* then propagated rostrally (*arrow* 2), while a decrease in the *red signal* was observed near the same point of origin (*arrow* a) and then propagated in the same direction (*arrow* b). There was complete loss of both *red* and *green signals* in this cell, consistent with full MF and AE. *B*, visualization of t*op-to-bottom* spatial progression of ACR and *bottom-to-top* progression of MF in the same sperm cell following addition of High P4 (+bicarb + CD). Before stimulation, the basal signal of the GCaMP3 is low, while maximal mCherry intensity is detected. Following stimulation, an increase in the GCaMP3 signal was detected near the *top* of the sperm head (*arrow* 1). The *green signal* propagated caudally toward the *bottom* of the sperm head (*arrow* 2), whereas a decrease in the *red signal* was observed at the *bottom* of the APM as soon as the *green fluorescence* intensity increased in that location (*arrow* a). The decrease in the red signal propagated rostrally toward the *top* of the sperm head (*arrow* b), resulting in a complete loss of both *red* and *green signals*. For both *A* and *B*, time is provided in seconds measured after the start of application of the stimulus, next to each frame. AcroSensE, Acrosome-targeted Sensor for Exocytosis; APM, acrosomal plasma membrane; CD, 2-hydroxypropyl β-cyclodextrin.

Live cell microscopy of AcroSensE sperm enabled us to determine the spatial characteristics of ACR and MF events. As seen in Figure 4, an *increase* in the fluorescent signal coming from the GCaMP3 was used to localize the initiation site of the intensity change associated with ACR, whereas the initiation site for AE was noted by the initial site of mCherry signal *loss*. Changes in fluorescence in both wavelengths were used to track the directionality and progression of Ca^2+^ rise and AE.

**Figure 5:**
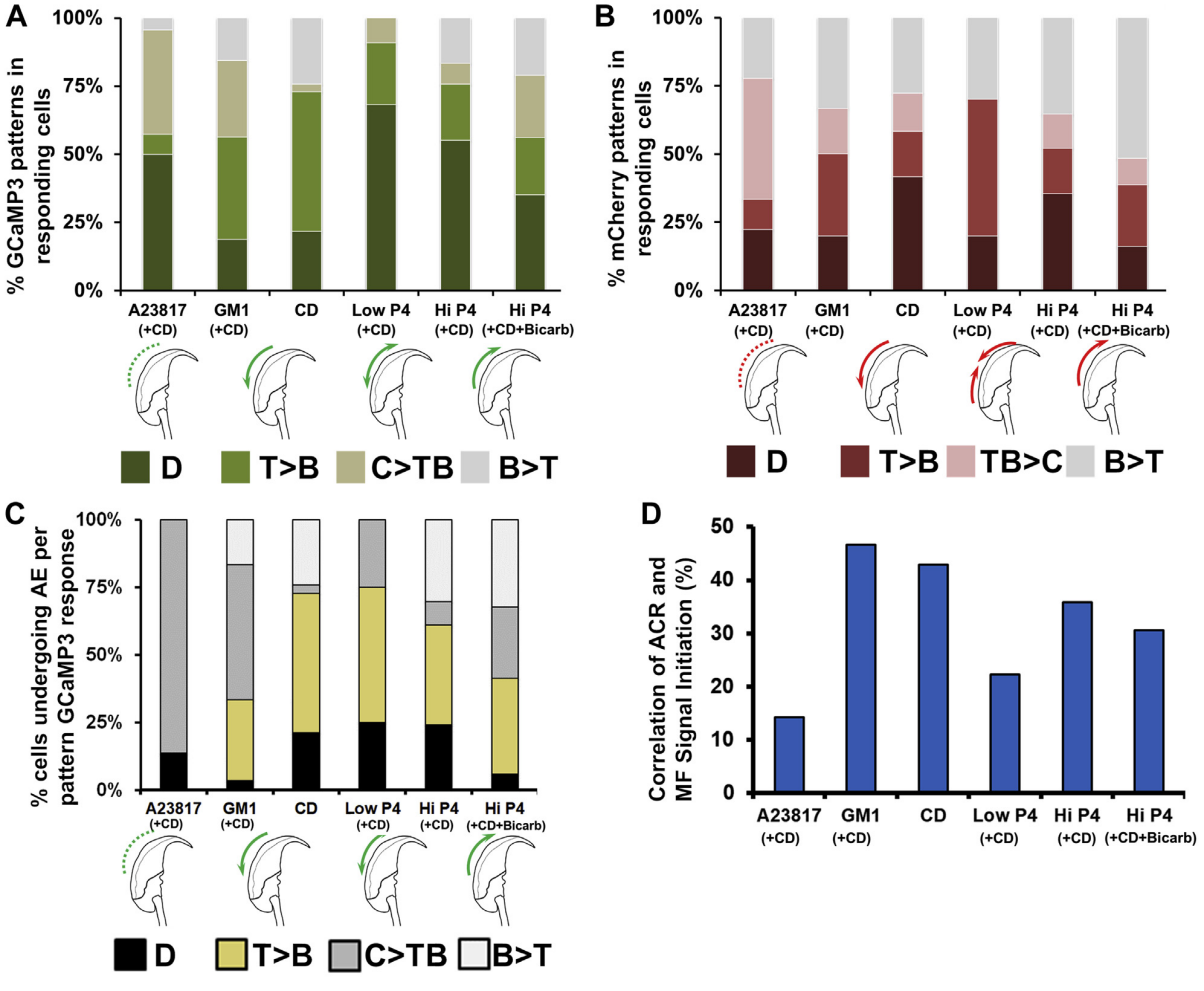
**Spatial characteristics of ACR and membrane fusion (MF) events.***A*, the percentage of cells in each experimental condition demonstrating diffuse (D), *top-to-bottom* (T > B), *center-to-top* and *center-to-bottom* (C > TB), and *bottom-to-top* (B > T) progression of ACR as reflected in GCaMP3 signal. All conditions were statistically different (*p* < 0.001 for all conditions excepting Low-P4/Hi-P4 *p* = 0.008). See Fig. S2 for values of all comparisons associated with this figure. *B*, the percentage of cells in each experimental condition demonstrating diffuse (D), *top-to-bottom* (T > B), *top-to-center* and *bottom-to-center* (TB > C), and *bottom-to-top* (B > T) progression of the membrane fusion (MF) events as indicated by the loss of the mCherry signal. All conditions were statistically different (*p* < 0.001 for all conditions excepting G_M1_/Hi-P4 *p* = 0.002), except for CD/Hi-P4 which were not significantly different (*p* = 0.39). *C*, summary of the percentage of cells that underwent AE following each of the spatially different Ca^2+^ rise progression patterns, under the various conditions. All conditions were statistically different with *p* < 0.001, except CD/Hi-P4 where *p* = 0.031. *D*, the percentage of cells in each experimental condition in which the MF and Ca^2+^ rise signals initiated at the same location. Note on nomenclature: in murine sperm, the apical acrosome is that portion that lies on the convex surface of the APM, so we use “rostral” or “*top*” to denote location near the perforatorium (tip) and “caudal” or “*bottom*” to denote location near the subacrosomal ring. ACR, acrosomal Ca^2+^ rise; AE, acrosome exocytosis; APM, acrosomal plasma membrane; CD, 2-hydroxypropyl β-cyclodextrin.

A summary of the findings for the spatial characteristics of ACR formation is provided in Figure 5*A*. We observed 4 main patterns of progression: (1) diffuse—in which the signal initiated evenly over the entire apical acrosome area (D), (2) top to bottom—initiating from a rostral position (“top” position, near the perforatorium [tip]) and progressing caudally (“bottom” position, near the subacrosomal ring) toward the postacrosomal region (T > B), (3) center outward—initiating at the center and propagating in opposite directions to the top and bottom of the apical acrosome (C > TB), and (4) bottom to top—progression rostrally from near the subacrosomal ring to the top (B > T). Following A23187 and various P4 stimulations, the plurality of responding cells showed diffuse patterns of Ca^2+^ rise throughout the acrosome. In sperm following the addition of exogenous G_M1_ or induction of sterol efflux by CD, this pattern was observed less frequently. Particularly, addition of G_M1_ or CD to cells resulted in increased incidence of signals initiating from the top or top/center (Fig. 5*A*). Initiation of ACR from the bottom of the acrosome near the subacrosomal ring was consistently lower under all conditions (16% of sperm when averaged across all conditions).

For MF (Fig. 5*B*), as determined by the loss of the mCherry signal, spatial patterns were similar with the distinction that instead of a signal propagating from the center to the ends as seen for ACR (C > TB pattern), we observed MF initiating from both ends and moving toward the center (bottom and top toward the center, TB > C). In cells incubated with bicarbonate during preincubation, we observed higher rates of B > T patterns of loss of the mCherry signal (>50% of responding cells for high P4 + CD + bicarbonate, Fig. 5*B*). In contrast, higher incidence of T > B were observed when P4 concentration (+CD) was low (Fig. 5*B*). All patterns were observed relatively equally upon G_M1_ stimulation, while addition of CD alone resulted in slightly less than half of the sperm demonstrating a diffuse pattern of release of the AcroSensE during AE (Fig. 5*B*).

Next we examined the percentage of cells transitioning from ACR to full MF and AE as a function of the initiation site of the rise in the AcroSensE fluorescence intensity (Fig. 5*C*). Interestingly, upon A23187 stimulation, ACR transformed into AE almost exclusively if the ACR events initiated at the center of the sperm head (C > TB). Under all conditions, cells were less likely to demonstrate progression from ACR to MF if the ACR events were diffusely localized or had a bottom-to-top progression. Addition of exogenous G_M1_ to the cells had a higher chance to generate MF (80% of cells) if the ACR events initiated at the center or the top of the sperm head, but not at the bottom (20% of cells). In the case of CD and low P4 with CD and bicarbonate, at least 50% of the sperm undergoing AE demonstrated ACR initiating rostrally (at the top of the sperm head) and moving downward toward the subacrosomal ring (CD: 51.5%, Low P4: 50%).

Finally, we looked at the spatial correlation between the initiation sites of ACR and MF. Figure 5*D* provides the percentage of cells in each condition where the ACR and MF initiated at the same location. Interestingly, in AcroSensE sperm stimulated with G_M1_ or CD, we see the highest rates of both ACR and MF demonstrating similar initiation sites (47% and 43%, respectively). P4 stimulation demonstrated somewhat lower rates of spatial correlation for ACR/MF initiation, up to 36% (for high P4). In contrast, A23187 demonstrated the lowest spatial correlation, with only 14% of the cells showing ACR and MF initiating at the same location. These data show that AE can initiate independently from the original site of ACR, but a stronger spatial link between that site and MF was associated in membrane lipid–mediated signaling.

### Temporal characterization of ACR events

**Figure 6:**
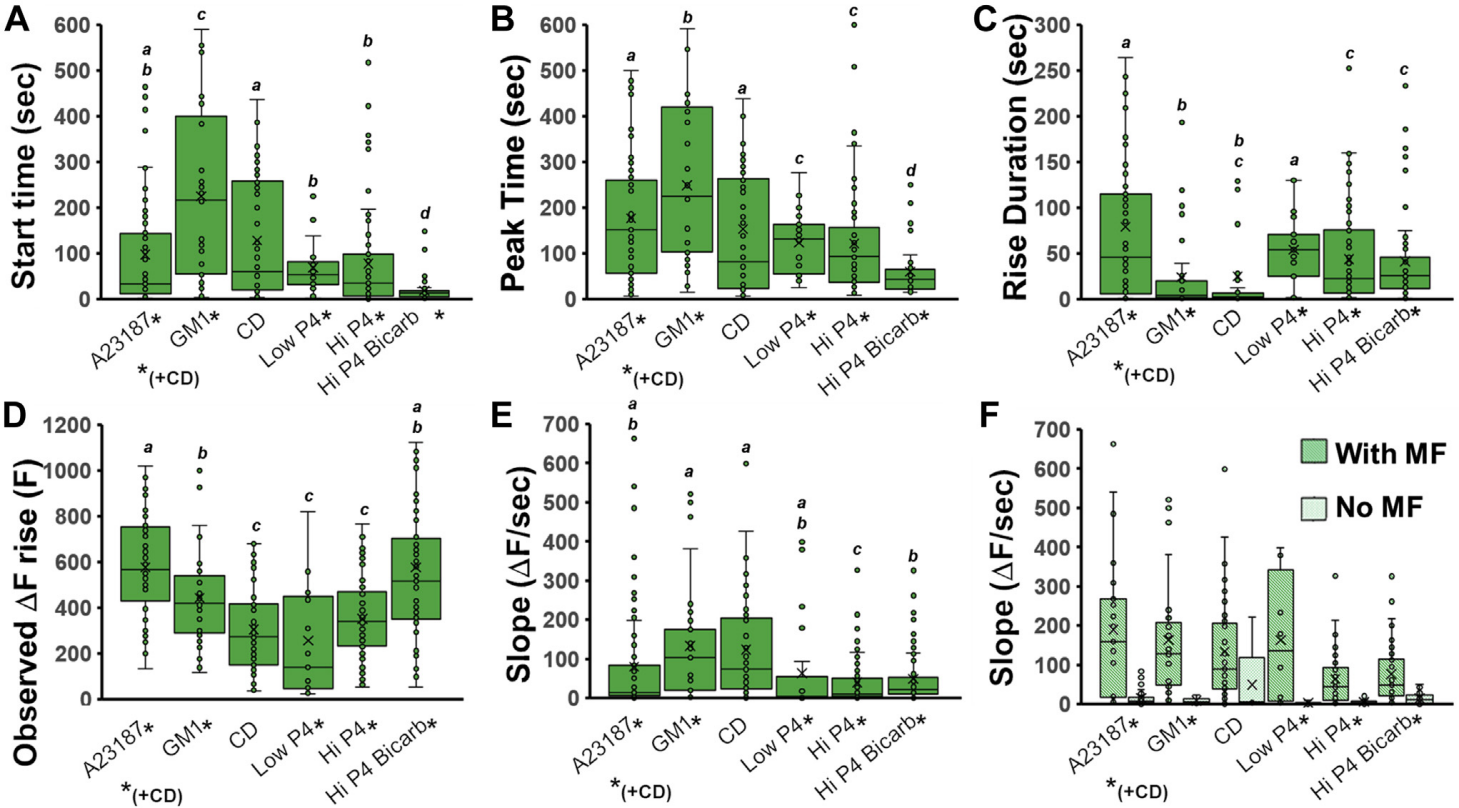
**Characterization of Ca**^**2+**^**rise upon various stimulations**. Statistical differences of *p* < 0.05 are noted by different letters. *A*, the average time from stimulation to the start of ACR. *B*, the average time of peak of the green GCaMP3 fluorescent signal. *C*, the average duration of the GCaMP3 signal rise, as calculated from the data presented in (*A*) and (*B*). *D*, the average total rise of the GCaMP3 fluorescent signal. *E*, the average rate of signal increase over time (slope) following stimulation. *F*, comparison of the slopes between cells that underwent membrane fusion and those that did not. ACR, acrosomal Ca^2+^ rise.

Next we examined key temporal characteristics of ACR as they occurred under various conditions (Fig. 6). Strikingly, we found substantial differences in the onset time of ACR depending on whether the stimulation was mediated by membrane lipids or P4. ACRs were significantly faster to appear following high P4 in sperm that had previously been capacitated with CD and bicarbonate (18.6 ± 3 s, Fig. 6*A*). Conversely, ACRs were slowest to initiate in sperm stimulated with exogenous G_M1_ (231 ± 31 s), more than 12 times slower. The trend of onset times between the different conditions was consistent with the values observed for time to peak, in which G_M1_-mediated ACR was the slowest and P4 was the fastest (Fig. 6*B*).

Surprisingly, these trends were followed in reverse regarding the total rise time (rise duration), in which longer start times and times to peak for CD and G_M1_ (Fig. 6, *A* and *B*) corresponded with shorter rise durations (Fig. 6*C*). The rise duration (Fig. 6*C*) indicates the timeframe in which the ACR either reached its peak value or diminished by the occurrence of AE. Comparable results were found for G_M1_ (18.7 ± 5.4 s) and CD stimulation (25.1 ± 12.4 s, Fig. 6*C*), whereas P4 demonstrated approximately 2-fold longer time-to-peak duration (Low P4+bicarb+CD: 54.4 ± 8.6 s, Hi P4+CD: 43.9 ± 5.1 s, Hi P4+bicarb+CD: 41.4 ± 6.3 s). Compared to all other conditions, the average time-to-peak duration was significantly longer following A23187 stimulation.

Significant differences of almost 2-fold were also observed between the various conditions for the GCaMP3 signal when looking at the rise amplitude (ΔF). Figure 6*D* summarizes the increase in the GCaMP3 fluorescence signal, which corresponds to the net change in the acrosomal Ca^2+^ concentration during ACR. Our data show that for A23187 (576 ± 20 ΔF) and High P4 with bicarbonate (577 ± 32 ΔF), the total Ca^2+^ rise in the acrosome was almost twofold higher than for CD (304 ± 32 ΔF, *p* < 0.001), or High P4 without bicarbonate during capacitation (352 ± 0.13 ΔF, *p* < 0.001), or Low P4 with bicarbonate (297 ± 44 ΔF, *p* < 0.001). GCaMP3 signal increase in response to G_M1_ was only marginally lower than the ionophore A23187 (*p* < 0.0081) or high P4 with bicarbonate (454 ± 43 ΔF).

Conversely, the kinetic rates of GCaMP3 Ca^2+^ signal rise (slope, ΔF/sec) were the highest following G_M1_ and CD stimulations (137 ± 25 ΔF/sec, and 124 ± 19 ΔF/sec, respectively), with other conditions being approximately two-fold slower (Fig. 6*E*). However, there was more cell-to-cell variation in this parameter reducing the statistical significance of many differences.

Remarkably, the kinetic rate of GCaMP3 fluorescent signal rise was directly associated with the ability of the stimulated sperm to undergo MF and AE. Figure 6*F* breaks down the slope (ΔF/sec) for the sperm in each condition that either underwent MF (AE) or did not. The data clearly show that although there was particularly high variance when stimulating with P4, in all conditions, cells that successfully transitioned from ACR to MF had significantly higher rates of GCaMP3 signal increase (*i.e.*, faster rates of Ca^2+^ influx into the acrosome).

### Identification and characterization of PSF-like events during AE

**Figure 7:**
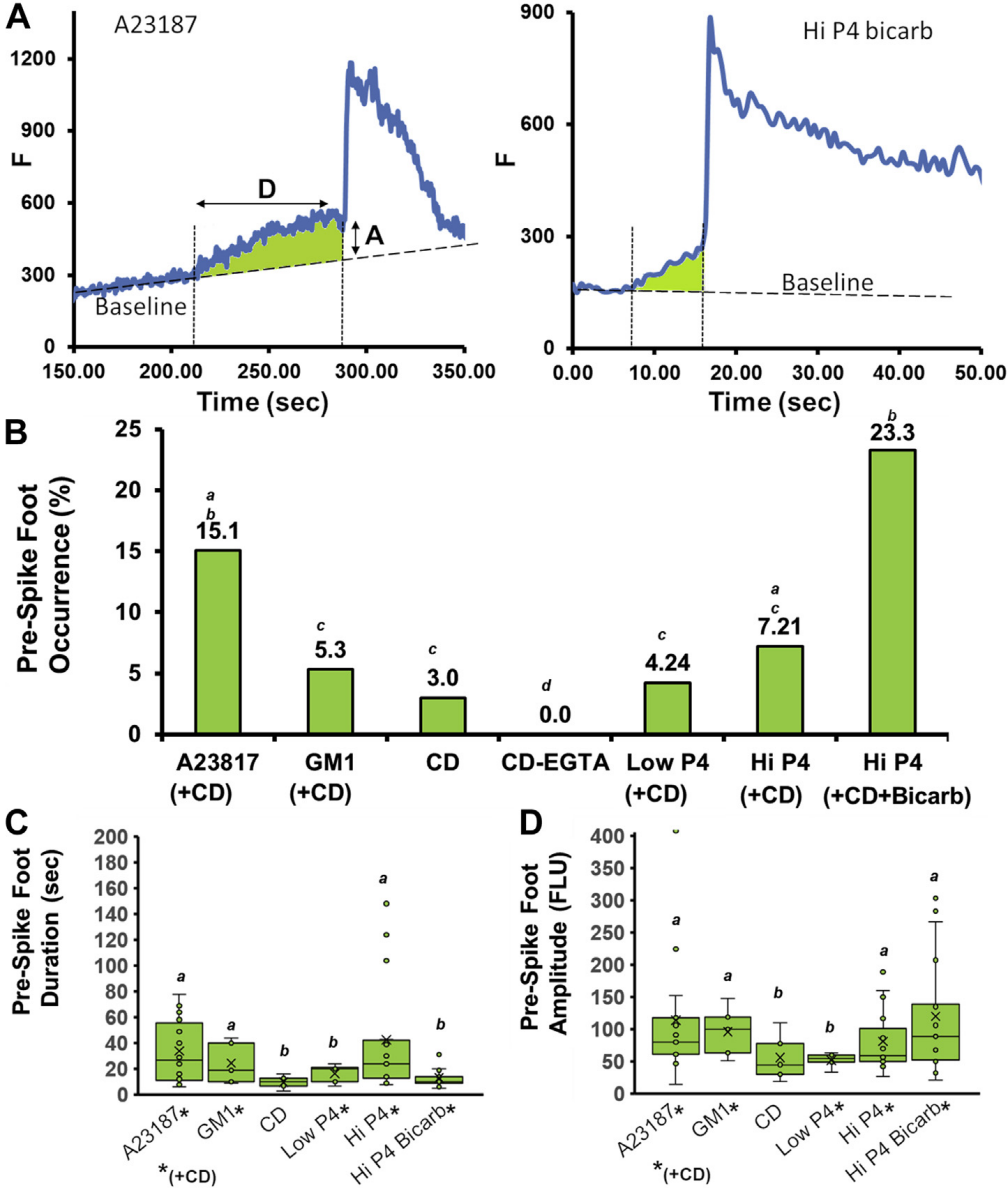
**Summary of prespike foot (PSF)–like events preceding ACR**. *A*, representative traces of PSF-like events following stimulations with A23817 (l*eft*) or high P4+bicarbonate+CD (*right panel*). The shaded area (*green*) is provided to highlight the PSF-like event. *Arrows* indicate the duration (*D*) and amplitude (*A*) above the baseline GCaMP3 signal, as measured for the analysis provided in (*C*) and (*D*). *B*, summary of PSF-like event occurrence (as the percentage of total cells) under various conditions. *C*, average duration of the PSF-like events, as measured from the initial rise of signal to the beginning of the main Ca^2+^ rise (see *left panel* in (*A*)). *D*, average amplitude of the fluorescence intensity increase during the PSF-like event. ACR, acrosomal Ca^2+^ rise.

Unexpectedly, the AcroSensE sperm revealed what is typically a subtle intermediate step in the process of MF and cellular exocytosis (Fig. 7). These events preceded the major Ca^2+^ rise (ACR), and while demonstrating much slower timescale, their occurrence and profile are reminiscent of PSF-like events observed in a variety of secretory cells when measured with amperometric methods using carbon fiber electrodes (44). Figure 7*A* provides two types of representative traces of PSF-like events detected in AcroSensE sperm, following stimulation with A23187 or high P4 (after incubation with CD and bicarbonate). The PSF-like events were defined as a Ca^2+^ signal rise that preceded the major ACR event and which demonstrated reduced amplitude and kinetics (*i.e.*, slope) in comparison to that ACR event. Figure 7*A* shows the application of these criteria for determining a PSF event, where the GCaMP3 signal rises above the baseline but demonstrates a distinctive dissimilar rise rate compared to the ACR. In addition, the PSF-like events were significantly smaller (4–7 fold) in amplitude than the ACR, depending on the condition, and resulted in a biphasic increase. Together, these two criteria were used to define these events. Except for sperm incubated in medium depleted of extracellular Ca^2+^ (EGTA in medium), PSF-like events were detected under all conditions, suggesting that these miniature elevations in the GCaMP3 signal were dependent on influx of extracellular Ca^2+^ into the acrosome. Nevertheless, considerably higher incidence of PSF-like events was observed in cells stimulated by A23187 (PSF detected in 15.1% of responding cells; *p* ≤ 0.024 *versus* G_M1_, CD and Low P4) and in cells stimulated with high P4 following capacitation in the presence of CD and bicarbonate (detected in 23.3% of responding cells; *p* ≤ 0.003 for G_M1_, CD and Low P4 and High P4, Fig. 7*B*).

Although their manifestation in response to high P4 stimulation was lower in the absence of bicarbonate, PSF-like event duration in this condition was roughly 3 times longer (42.5 ± 4.6 s) than that of low P4 or high P4 with bicarbonate (16.4 ± 1.6, and 13.1 ± 1 s, respectively; Fig. 7*C*). The amplitude values of the PSF-like events (as measured from the baseline cell fluorescence to the amplitude of where the foot transformed into a typical ACR slope) were independent of the duration parameters, demonstrating the highest values in cells stimulated with high P4 with bicarbonate and in cells stimulated with ionophore A23187 (Fig. 7*D*). Nonetheless, by definition, the average amplitude was markedly lower than the amplitude of ACR (4–7 fold), indicating the limited amount of Ca^2+^ ions able to enter the acrosome *via* the PSF-like events.

### Characterization of MF dynamics in the AcroSensE sperm

**Figure 8:**
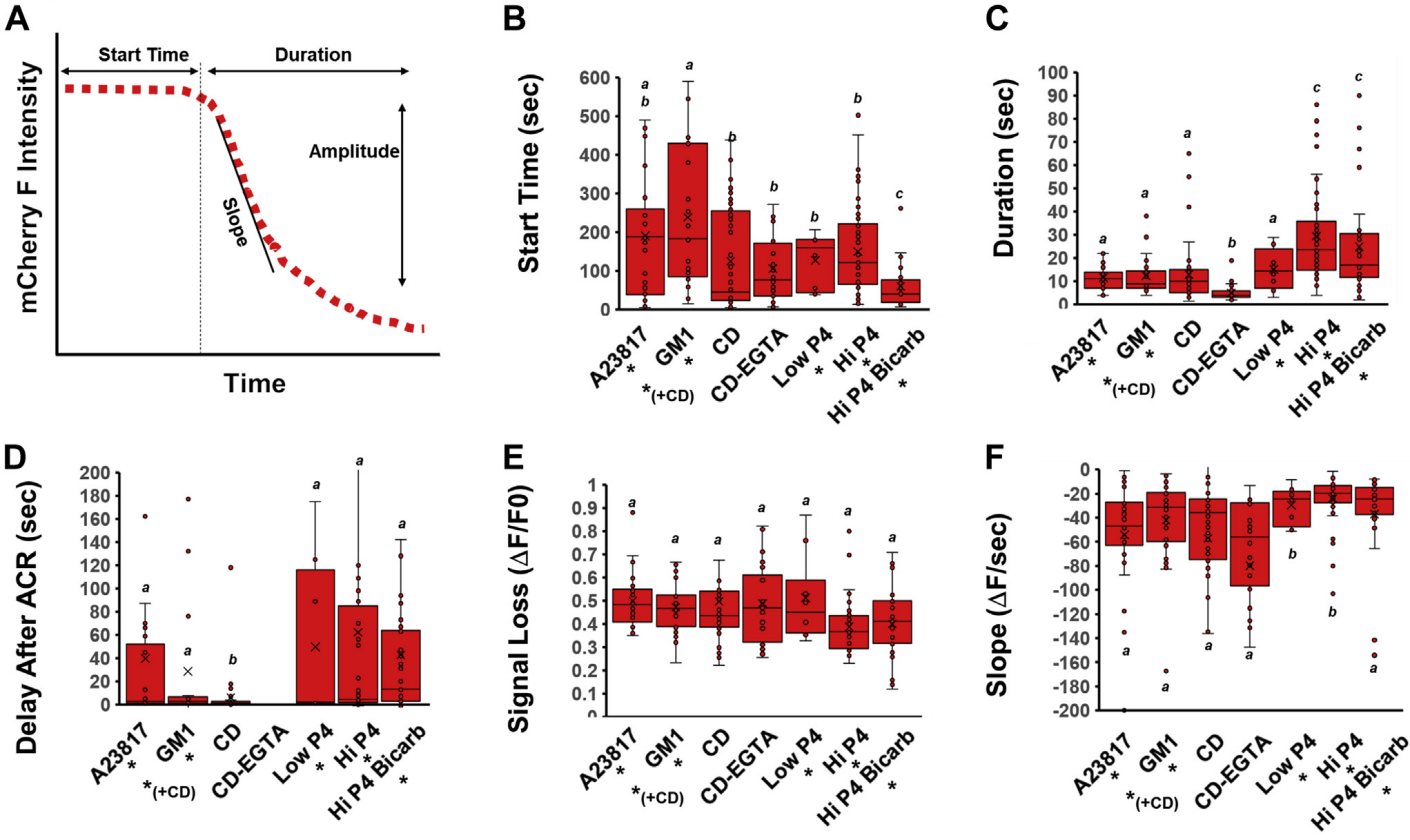
**Characterization of membrane fusion (MF) events under various conditions**. Statistical differences of *p* < 0.05 are noted by different letters. *A*, a schematic illustration of typical MF proceeding until full AE as detected from the loss of mCherry fluorescence in the acrosome and the various parameters used in the analysis provided in (*B*–*F*). *B*, the average time in which MF initiated, as measured from the time of stimulation to the start of loss of the mCherry signal. *C*, the average duration of the mCherry fluorescence loss until it reached its minimum intensity. *D*, the average delay between the onset of the ACR (initial rise in *green* GCaMP3 signal) and the initiation of MF (mCherry signal loss). Compared to all other conditions, the delay between ACR and MF was the shortest for CD, with *p* values of 0.06 (A23187), 0.03 (G_M1_), 0.01 (*Low* P4), 0.0003 (Hi P4), and 0.0005 (*High* P4 + bicarb). *E*, the average change in the mCherry signal during acrosome exocytosis (ΔF), normalized to the baseline fluorescence intensity (F0). *F*, the rate of signal loss over time as calculated from the slope of the mCherry fluorescence decay (as measured from the initiation of signal loss). ACR, acrosomal Ca^2+^ rise; AE, acrosome exocytosis; CD, CD, 2-hydroxypropyl β-cyclodextrin.

In mice expressing GFP in the acrosome, loss of fluorescence results from loss of acrosomal matrix contents due to MF events (45). As shown in the aforementioned figures, we used a similar strategy to relate loss of mCherry fluorescence and MF events and full AE. In Figure 8*A*, we schematically show several analyses of the loss of the mCherry signal we performed to determine whether they differed with various forms of stimulation. First, we determined the onset of MF events following the various stimulations. As shown in Figure 8*B*, MF initiated nearly 2.5 times faster in response to high P4 in sperm that were capacitated with CD and bicarbonate *versus* those that were incubated only with CD (60 ± 11 and 149 ± 16 s, respectively, *p* = 4.9 × 10^−5^ Fig. 8*B*). In contrast, following the addition of G_M1_ or A23187, cells demonstrated the longest delay to the onset of the MF responses (238 ± 38 s and 190 ± 29 s, respectively, Fig. 8*B*). Note that the trends of start times between the different conditions were comparable for ACR and MF.

Next, we determined the duration of MF events, as calculated from the time frame of the start of mCherry signal loss to complete loss of signal, consistent with the completion of AE. Interestingly, cells stimulated with CD under zero Ca^2+^ conditions demonstrated the shortest duration times of the mCherry signal dispersal from the acrosome (CD-EGTA, 5 ± 0.6 s). On the other hand, stimulation of cells with P4 resulted in the longest duration periods regardless of the presence or absence of bicarbonate during capacitation (29.41 ± 2.69 and 24.87 ± 3.60 s, respectively; Fig. 8*C*). All other conditions demonstrated similar times for the diffusion of the AcroSensE out of the acrosome ranging from 11 to 15 s on average.

As a single fusion protein comprising both the GCaMP3 and mCherry elements, the AcroSensE construct also allowed us to look at the delay time between ACR and full MF. Figure 8*D* summarizes the ACR–MF time interval, as calculated from the time between the onset of the ACR (start of increase in GCaMP3 signal) and the initiation of the MF (when the mCherry signal starts to decrease). The ACR–MF time interval showed little variability among the various conditions, except for cells stimulated with CD where the delay between Ca^2+^ rise and MF was considerably shorter than all other conditions (6.1 ± 3 s). Importantly, no significant differences were observed for the total amount of mCherry fluorescent signal lost as demonstrated by the ΔF/F_0_ analysis provided in Figure 8*E*, suggesting that under all conditions tested here, once AE occurred, the loss of protein content (matrix) from the acrosome vesicle was to a similar extent. Conversely, the rates of protein diffusion out of the acrosome during AE were dependent on the stimulation, where P4 signaling resulted in slower protein release rates and lipid modulation of the sperm or addition of A23187 induced generally faster rates of protein diffusion out of the matrix within the acrosome vesicle (Fig. 8*F*).

## Discussion

Sperm from the AcroSensE mice demonstrated spatiotemporal relationships between Ca^2+^ dynamics in the compartment of the acrosome and MF between the plasma membrane and OAM. Differences in response to varied stimuli in turn revealed important insights into the mechanisms and pathways leading to AE, enabling us to generate a working model (shown below) that summarizes current understanding.

### Mechanistic findings

Working through the findings as they would occur temporally during AE, data presented here show absolute reliance on extracellular Ca^2+^ for ACR. Interestingly, some exocytosis was observed under (nonphysiologic) Ca^2+^-depleted conditions. These were the only conditions under which MF occurred without ACR. This observation could be explained by some combination of two known mechanisms that are not physiologically relevant: EGTA causes membrane ruffling/perturbation (46), and Ca^2+^-independent PLA_2_β has been shown to be responsible for spontaneous (unstimulated) AE (47).

Based on the current understanding that most sperm undergo AE before they reach the zona pellucida (which had been the main stimulus in the acrosome reaction model) (6, 7, 48), we focused our attention on stimuli that sperm would encounter in the vicinity of the egg (progesterone from cumulus cells), that would mimic stimuli found in the oviduct in a reductionist, controlled way (CD as a chemically defined mediator of sterol efflux replacing effects of oviductal albumin or high-density lipoproteins), or stimuli that exist on sperm but are released from inhibition provided by the binding of seminal plasma proteins (G_M1_). The methodological soundness of delivering these stimuli *via* puffing by micropipette was supported by the similarity of results with puffed CD to those seen when we preincubated sperm with CD in the medium (Fig. S1). Also, current results with CD and G_M1_ closely matched those when incubating sperm in media in prior studies (23). One interesting finding in making these comparisons within and between studies is that with membrane lipid–stimulated exocytosis, it appears that a subpopulation of roughly 45 to 50% of sperm can maximally be triggered to undergo AE. This finding is consistent with a wealth of data across species that only a subset of sperm can undergo physiologically relevant capacitation and AE.

#### How does extracellular Ca^2+^ increase acrosomal Ca^2+^?

Our data suggest that PSF-like events and ACR are most likely mediated by FP formation between the APM and OAM bilayers, as evidenced *via* the increase in GCaMP3 signal mediated by Ca^2+^ flux into the acrosome. A limited number of FPs, FPs of a transient nature, and/or FPs of very small diameter could all account for the PSF-like events. As the number/size of FPs would increase, this would lead to more rapid ACR, and simultaneously the increase in MF events would be predicted to result in quicker AE. This relationship would account for our finding that the rate of ACR in sperm undergoing MF/AE was higher than that in cells that did not demonstrate MF (Fig. 6*F*). However, mechanisms for Ca^2+^ entry into the acrosome other than FPs must also be considered:

1. *GCaMP3 signal increase was a result of Ca*^*2+*^*elevation in the acrosome generated by mobilization from other internal stores.* Mobilization of Ca^2+^ ions from one of the sperm’s internal stores (*i.e.*, mitochondria or redundant nuclear envelope [RNE] (49)) into the acrosome would result in an increase in the acrosome Ca^2+^ concentration, without involvement of Ca^2+^ influx *via* FPs from the medium. However, this alternative can be ruled out by data showing that GCaMP3 signals were absent when stimulating cells in Ca^2+^-depleted medium (EGTA), demonstrating the dependence of the acrosomal GCaMP3 signal on extracellular Ca^2+^. Moreover, this alternative is also spatially incompatible with our observations, as both the putative Ca^2+^ stores in the RNE and mitochondria are located in the connecting piece/midpiece of the sperm, whereas we frequently observed high occurrence of ACR initiating at the top (apical tip) or center of the sperm (Fig. 5*A*).
2. *The ACR was mediated via transporters and/or channels localized on the OAM.* In this alternative model, Ca^2+^ entry to the acrosome occurs in a two-step process, where cytosolic Ca^2+^ increase (*via* channels/transporters on the APM or release from RNE/mitochondria stores) is followed by Ca^2+^ entry into the acrosome through either a channel or a transporter on the OAM. Indeed, several proteins have been reported to be involved in acrosome Ca^2+^ mobilization including Ip3R (50) and TPC1/TPC2 (NAADP-gated intracellular Ca^2+^/Na^+^ channels (51, 52)). Because of its baseline high Ca^2+^ concentration, additional influx into the acrosome of a mature sperm must occur against the electrochemical gradient, requiring expenditure of energy. Two mechanisms that could function in this capacity have been reported to enable Ca^2+^ influx into the acrosome. First, sarcoplasmic/endoplasmic reticulum Ca^2+^-ATPase 2 (SERCA2) has been shown to be expressed in mammalian sperm (53) and regulate Ca^2+^ uptake into the acrosome (41). There are conflicting data regarding SERCA activity in mature sperm, with functional effects from inhibition by thapsigargin tending to occur only at high concentrations. Furthermore, studies in HeLa cells showed that intracellular alkalinization inhibits SERCA (54), suggesting that in capacitated sperm, these pumps would demonstrate lower activity. Alkalinization of the acrosome itself has been observed during capacitation and is also observed throughout the cytoplasm. Second, the secretory pathway Ca^2+^ ATPases (SPCAs) have been reported to function in mammalian sperm (49). Although SERCA or SPCA pump activation under some conditions could result in ACR, this rise would be predicted to be independent from extracellular Ca^2+^ [*e.g.*, refer to figure 2 in the study by Williams and Ford (55)]. In addition, in human sperm, the SPCA clearly localized to the midpiece/connecting piece, and not the acrosome. Together with our data using EGTA, these transporters seem unlikely to explain ACR events in the AcroSensE sperm. In addition, based on reports of Ca^2+^ clearance from mouse sperm (which involves all the available Ca^2+^ transport mechanisms (56)), it is clear that the theoretical velocity and rate of Ca^2+^ influx into the ER as mediated by this channel are slower than we observe during ACR. Yet another candidate for mobilizing Ca^2+^ into the acrosome is the stromal interaction molecule (STIM)–Orai channel complex involved in store-operated Ca^2+^ entry (49, 57, 58, 59). Indeed, STIM and Orai proteins were documented in human (49) and mouse sperm (60) by Western blot and immunocytochemistry. Using cell-penetrating peptides attached to a protein domain that stimulates STIM–Orai activity, Morris *et al* (61) were able to induce store-operated Ca^2+^ activity at the neck of human sperm, downstream of CatSper stimulation by progesterone. Thus, these findings strongly suggest that as with SPCA, this store-operated Ca^2+^ entry complex is spatially separated from our observations of Ca^2+^ increase located over the acrosome/apical-acrosome during ACR.
3. *Change in acrosomal pH positively modulates the GCaMP fluorescence intensity.* According to this alternative explanation, pH changes in the acrosome would increase GCaMP3 fluorescence, mimicking a true ACR and leading us to interpret the results as FP formation. Before capacitation, the acrosomal pH is maintained at acidic levels *via* several mechanisms (including V-ATPase, sodium–proton exchanger (NHE), and Cl^−^/HCO3^−^ exchanger (62)). During capacitation, the acrosomal pH was shown to gradually increase over the incubation period of 120  minutes from pH 5.3 ± 0.1 to pH 6.2 ± 0.3 (63). Thus, the pH increase in the acrosome is occurring too slowly to explain the rapid changes in GCaMP3 fluorescence intensity observed for the AcroSensE sperm during FP formation. Moreover, it is also important to note that the GCaMP Ca^2+^ indicator is relatively stable in the physiological pH range that has been reported for the acrosome (64), and therefore, abrupt fluorescence intensity changes as seen during our recordings are highly unlikely to be a result of pH fluctuations.
4. *Release of sequestered Ca*^*2+*^*from Ca*^*2+*^*-binding proteins.* Several types of Ca^2+^-binding proteins have been detected in spermatozoa, including calmodulin, calreticulin, CABS1, and glucose-regulated protein 78 (BiP), although the latter demonstrates only low Ca^2+^-binding capacity (65). These proteins have been reported to be localized throughout the sperm head and flagellum, but little is known about Ca^2+^-binding protein functions in the acrosome. Ca^2+^-binding proteins typically release bound Ca^2+^ ions upon depletion of a store, which would not be observable with the AcroSensE mice.

#### How does ACR trigger MF/AE?

Our results showed clearly that it was the rate of ACR, and not how long it takes to induce it or the absolute amounts of Ca^2+^ brought into the acrosome, that was most clearly linked with ability to undergo MF/AE (Fig. 6). This was seen with both the nonphysiological ionophore A23187, in which slow rise led to very high GCaMP3 fluorescence but relatively few cells showing MF, as well as with the physiologically relevant stimuli. At least two separate mechanisms could account for the existence of these subpopulations. First, the sperm exhibiting slow rise in response to A23187 might have had that increase mediated by different processes (*e.g.*, A23187 permeating through APM and then the OAM, which would result in Ca^2+^ leaking into the acrosome), which were not coupled to mechanisms promoting MF/AE. Second, this subpopulation of cells might have had other differences that decoupled acrosomal Ca^2+^ concentrations and MF/AE. For example, differences in membrane lipid composition such as in local sterol abundance could render them less fusogenic. Regardless, the retention of mCherry signal showed that AE was not triggered in these cells. Under all conditions, the rate of ACR was much higher in sperm that underwent MF/AE than in sperm that did not (Fig. 6*F*). Whether the rate of ACR itself regulates MF such as through stimulation of MF machinery (*i.e.*, SNARE proteins) or whether a faster rate of ACR results from pre-existing membrane differences that would have made them more fusogenic cannot be determined by these correlations and will be a topic of future study.

Interestingly, the AcroSensE sperm also revealed that the spatial location of the initiation site of ACR and initiation site of MF tended to vary with the stimuli (Figs. 5 and S2). This finding is of importance because it suggests that the stimuli exerted their effects through different, functionally redundant mechanisms that reach a common endpoint. However, the existence of different patterns in response to a single stimulus is evidence that there is not a single initiation site or spatial pathway for propagation of ACR or for MF that is unique for each stimulus. A biological explanation for such differences could be that plasma membrane proteins that mediate sterol efflux (such as ATP-binding cassette transporters) or act as receptors for P4 are enriched in certain membrane subdomains, but these are distributed throughout the plasma membrane over the apical acrosome. Biophysical factors such as local differences in membrane fluidity and distances between phospholipid head groups in the highly curved apical acrosome could lead to differences in ease of integration of lipids such as G_M1_, or the removal of sterols. The architecture of the sperm head and localization of microdomains highly enriched in sterols can also lead to mechanical resistance to fusogenicity (66).

Mechanistically, some differences in the patterns and kinetics of ACR can be explained by the hypothesized mechanisms of action for each stimulus. For example, G_M1_ took the longest time to initiate ACR and time to peak (Fig. 6, *A* and *B*). This would make sense if this sphingolipid acted as previously proposed, by inserting into the plasma membrane and then causing a focal enrichment, leading to a transient that would then locally prime MF machinery, facilitating AE in response to a subsequent Ca^2+^ wave (23). In contrast, CD had a shorter time to ACR and the shortest delay between initiation of ACR and MF (Fig. 8*D*). This would be consistent with a mechanism of action involving activation of PLB, which would cause immediate local changes in membrane curvature through its hydrolysis of both fatty acid tails at the sn-1 and sn-2 sites, leading to MF events (22). Both of these stimuli function by affecting the local membrane lipid environment, and it is noteworthy that both had the highest transition rates (the percentage of sperm that exhibited ACR progressing to MF/AE; Fig. 3*C*). If broken out by suppopulation, those sperm responding to A23187 with a fast ACR had a similar transition percentage to G_M1_ and CD as noted earlier in the study.

### Findings lead to a model for AE

Across all stimulation conditions excepting the small subset of cells responding nonphysiologically in the presence of EGTA, ACR was required for, and preceded, MF and AE. Of critical importance in trying to resolve existing controversy over the process of AE, the AcroSensE model enabled moderately fast imaging with high resolution. Spatiotemporal analysis of rise in GCaMP3 signal and loss of mCherry fluorescence *surprisingly showed a lack of correlation between the sites of initiation of ACR and* MF. G_M1_ and CD showed the highest levels of correlation, but even these were below 50%. This very simple finding provides compelling evidence against the acrosome reaction model in which fusion between the plasma and OAMs results in explosive exocytosis akin to popping a water balloon.

Rather, we observed that different stimuli were more likely to initiate ACR in different parts of the apical acrosome and therefore have that rise in Ca^2+^ propagate in different directions (Figs. 5, and S3). These data support a model in which FPs can be initially formed over the apical acrosome in different places. From that initiation site, elevated Ca^2+^ is soon found throughout the acrosomal matrix, and the presence of FPs large enough to allow outward diffusion of the AcroSensE protein can begin to occur at a distance within the apical acrosome.

**Figure 9:**
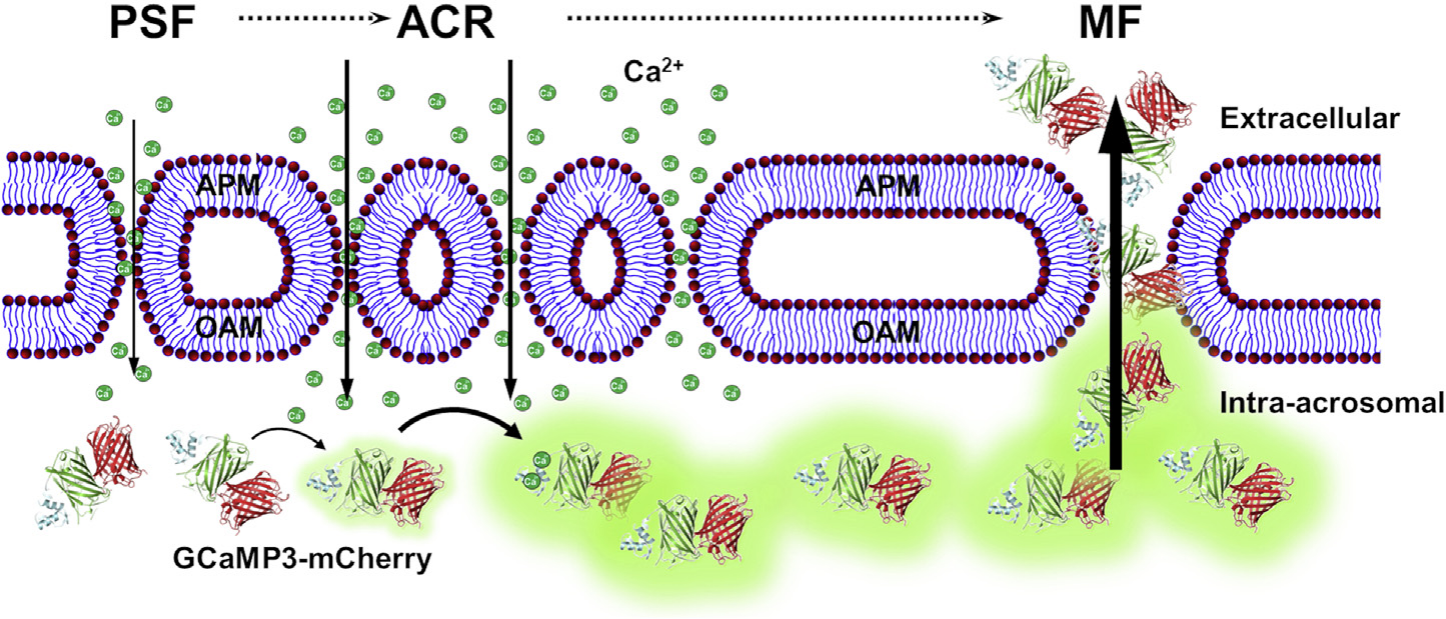
**Schematic illustration of hypothesized changes in AcroSensE fluorescence, Ca**^**2+**^**flux, and membrane fusion (MF) events between the plasma membrane overlying the acrosome (APM) and outer acrosomal membrane (OAM) of the sperm head.** Prespike foot (PSF)–like events occur as a result of transitory membrane fusions, either before or leading to more stable—but still small—fusion pores (FPs). These small focal fusion events enable Ca^2+^ influx into the acrosome. Full-membrane fusion leading to the loss of mCherry signal and the loss of acrosomal contents occurs as a result of coalescence of FPs. In this model, the dimensions of the FPs initially allow only for influx of small molecules into the acrosome’s lumen, including extracellular Ca^2+^ ions that can bind to the GCaMP3, resulting in an increase in the “*green*” fluorescence intensity (ACR). Progression of the FP into full-membrane fusion events results in much larger openings resulting from the loss of hybrid APM/OAM membrane vesicles, allowing the AcroSensE protein to diffuse out of the acrosome lumen into the extracellular space, resulting in a decrease in both the “*red*” and “*green*” fluorescence intensities. ACR, acrosomal Ca^2+^ rise; APM, acrosomal plasma membrane; OAM, outer acrosomal membrane.

The most parsimonious explanation for all our findings combined is shown as a model in Figure 9. In this model, we feature the MF events that result in changes in AcroSensE fluorescence. More complexity can of course be added in terms of MF machinery, plasma membrane channels, membrane microdomains, etc. In the model, transitory MFs can occur leading to the small increases in acrosomal Ca^2+^ that appear similar to either small FPs or small FPs are formed as a result of the same stimuli. The small size of the FPs is implied by the fact that the increase in GCaMP3 is seen before any mCherry is lost, suggesting that small molecules such as Ca^2+^ can enter before something the size of mCherry in fusion with the GCaMP3 (approx. 75 kDa) can leak out. Over time, the number of FPs increases in response to the one or more stimuli, and eventually, they coalesce to make hybrid vesicles resulting in full AE. Note that although our data (Fig. 8*E*) show loss of mCherry signal occurring to the same degree under all conditions, both the length of time over which it is lost (Fig. 8*C*) and the rate at which it is lost (Fig. 8*F*) differ between the membrane lipids and A23187 and the progesterone treatments. Given that the fusion construct is a soluble protein, this difference in the rate of loss likely reflects differences in the size and/or number of FPs through which the protein would diffuse out.

Overall, the combined data provided by the AcroSensE mice clearly support a regulated, multistep model of AE instead of the prior dogma of the acrosome reaction. Although AE is a common endpoint, different stimuli were found to induce it with different spatial and temporal dynamics underscoring functional redundancy in mechanisms for this critical step, which is essential for the transmission of life.

## Experimental procedures

### Reagents and animals

All reagents were purchased from Sigma, unless otherwise noted. Male and female B6129SF/J mice were purchased from Jackson Laboratories. All animal procedures were performed under the guidelines and approved by the Institutional Animal Care and Use Committee at Cornell University.

### Acr-GCaMP3-mCherry plasmid construction

Plasmid construction was performed by BlueSky BioServices. The pAcr3-EGFP backbone plasmid was a gift from Dr Masahito Ikawa (Osaka University). The GCaMP3-expressing vector (#63885) and mCherry were obtained from Addgene. The mCherry was first inserted downstream of the GCaMP3, and then the GCaMP3–mCherry cassette was inserted into the Acr-EGFP plasmid (pBluescript II SK+), which had had the GFP portion excised using EcoRI. The full construct contained a 2.4-kb mouse genomic region carrying the acrosin gene with the sequences of a signal peptide and an N-terminal peptide (KDNTT). The plasmid was verified by sequencing using the following primers: GTGGAGCATTGTGAGGTCACAG and GAGCTTGCCGGTGGTGCAGATG.

### Transgenic mice

Acr-GCaMP3-mCherry transgenic mice were generated by microinjection of gel-purified linearized DNA into a pronucleus of 100+ fertilized embryos collected from superovulated mice using standard techniques. Embryos for injection were obtained by mating (C57BL6/J and CBA) F1 hybrids. Transgenic founders were backcrossed to C57BL/6 mice. Primers for genotyping and RT-PCR were GTGGAGCATTGTGAGGTCACAG and GAGCTTGCCGGTGGTGCAGATG. Immunoblotting was performed on tissues collected from the AcroSensE mice including the brain, heart, muscle, testis, and liver. Protein was quantified, and equal amounts of protein were loaded for SDS-PAGE. Blots were blocked with 10% milk overnight at 4 °C. The primary rabbit GFP antibody (Abcam) was diluted 1:2500 in Tween Tris buffered saline to 2.5 μg/μl and incubated for 2 h at room temperature. Anti-rabbit IgG linked to horseradish peroxidase was used as a secondary antibody (Species-Specific F(ab’)2 fragment from donkey; GE Healthcare) and was diluted 1:3000 in Tween Tris buffered saline and incubated at 4 °C overnight.

### Live sperm Ca^2+^ imaging

For murine sperm, a modified Whitten’s medium (MW; 22 mM Hepes, 1.2 mM MgCl_2_, 100 mM NaCl, 4.7 mM KCl, 1 mM pyruvic acid, 4.8 mM lactic acid hemi-Ca^2+^ salt, pH 7.35; (67)) was used for all incubations. Glucose (5.5 mM), NaHCO_3_ (10 mM), and CD (3 mM) were supplemented as needed. Cauda epididymal murine sperm collection was described previously (68). All steps of collection and washing were performed at 37 °C using modified MW medium, using methods to minimize membrane damage.

For microscopy, sperm were plated on poly-D-lysine–coated 35-mm coverslip dishes (MatTek Corp), and 3 ml of warm MW base media supplemented with 10 mM CaCl_2_ was added. The dish with the sperm was then mounted on a Zeiss 510 microscope with a heated 100X oil immersion objective (37 °C). GCaMP3 was excited using the 488-nm line of a krypton/argon laser and viewed with a 505− to 550-nm BP filter. The mCherry was excited using the 555-nm line of a HeNe laser and viewed with a 575− to 615-nm BP filter. Selected cells were approached with a ∼5-μm-diameter pulled-glass capillary, prefilled with stimulating solution, and positioned within 100 μm from the sperm head. The stimulating solutions were made at 5X the normal concentration (*e.g.*, G_M1_ = 125 μM) to compensate for diffusion in the volume/space between the capillary and the cell. Sperm cells were imaged at 2 to 4 Hz while applying a puff of 10 s/5 psi from the pipette, controlled by a Picospritzer III (FMI Medical Instruments). Unless otherwise stated, all data are presented as mean ± SEM. For each of the experiments, the number of cells analyzed (n) is presented in the text or legend. Data were processed and plotted using Origin 8 (OriginLab) and Excel (Microsoft). Statistical comparisons (Student’s *t* test or χ^2^) were performed using Excel or JMP Statistical Software and are provided in Figs. S2–S7.

### Image analysis

Offline image analysis was conducted using Zeiss LSM image analysis software and ImageJ (National Institutes of Health). Changes in fluorescence (F) were normalized by the initial fluorescence (F0) and are expressed as ΔF.

## Data availability

The data that support the findings of this study are available from the corresponding author upon reasonable request.

## Supporting information

This article contains supporting information.

## Conflict of interest

A. J. T. discloses that he is a founder and an officer of Androvia LifeSciences, LLC, a biotechnology company investigating solutions for male infertility, in which he holds a minor equity stake. There are no conflicts with the current work. All other coauthors declare that they have no conflicts of interest with the contents of this article.

## References

[1] Kim K.S., Cha M.C., Gerton G.L. (2001). Mouse sperm protein sp56 is a component of the acrosomal matrix. Biol. Reprod..

[2] Kim K.S., Gerton G.L. (2003). Differential release of soluble and matrix components: Evidence for intermediate states of secretion during spontaneous acrosomal exocytosis in mouse sperm. Dev. Biol..

[3] Jin M., Fujiwara E., Kakiuchi Y., Okabe M., Satouh Y., Baba S.A., Chiba K., Hirohashi N. (2011). Most fertilizing mouse spermatozoa begin their acrosome reaction before contact with the zona pellucida during *in vitro* fertilization. Proc. Natl. Acad. Sci. U. S. A..

[4] Baibakov B., Gauthier L., Talbot P., Rankin T.L., Dean J. (2007). Sperm binding to the zona pellucida is not sufficient to induce acrosome exocytosis. Development.

[5] Hino T., Muro Y., Tamura-Nakano M., Okabe M., Tateno H., Yanagimachi R. (2016). The behavior and acrosomal status of mouse spermatozoa *in vitro*, and within the oviduct during fertilization after natural mating. Biol. Reprod..

[6] La Spina F.A., Puga Molina L.C., Romarowski A., Vitale A.M., Falzone T.L., Krapf D., Hirohashi N., Buffone M.G. (2016). Mouse sperm begin to undergo acrosomal exocytosis in the upper isthmus of the oviduct. Dev. Biol..

[7] Bhakta H.H., Refai F.H., Avella M.A. (2019). The molecular mechanisms mediating mammalian fertilization. Development.

[8] Selvaraj V., Buttke D.E., Asano A., McElwee J.L., Wolff C.A., Nelson J.L., Klaus A.V., Hunnicutt G.R., Travis A.J. (2007). GM1 dynamics as a marker for membrane changes associated with the process of capacitation in murine and bovine spermatozoa. J. Androl..

[9] Moody M.A., Cardona C., Simpson A.J., Smith T.T., Travis A.J., Ostermeier G.C. (2017). Validation of a laboratory-developed test of human sperm capacitation. Mol. Reprod. Dev..

[10] Cardona C., Neri Q.V., Simpson A.J., Moody M.A., Ostermeier G.C., Seaman E.K., Paniza T., Rosenwaks Z., Palermo G.D., Travis A.J. (2017). Localization patterns of the ganglioside GM1 in human sperm are indicative of male fertility and independent of traditional semen measures. Mol. Reprod. Dev..

[11] Schinfeld J., Sharara F., Morris R., Palermo G.D., Rosenwaks Z., Seaman E., Hirshberg S., Cook J., Cardona C., Ostermeier G.C., Travis A.J. (2018). Cap-score prospectively predicts probability of pregnancy. Mol. Reprod. Dev..

[12] Grafen A., Schumacher F., Chithelen J., Kleuser B., Beyersdorf N., Schneider-Schaulies J. (2019). Use of acid ceramidase and sphingosine kinase inhibitors as antiviral compounds against measles virus infection of lymphocytes *in vitro*. Front. Cell Dev. Biol..

[13] Molina L.C.P., Gunderson S., Riley J., Lybaert P., Borrego-Alvarez A., Jungheim E.S., Santi C.M. (2019). Membrane potential determined by flow cytometry predicts fertilizing ability of human sperm. Front. Cell Dev. Biol..

[14] Sharara F., Seaman E., Morris R., Schinfeld J., Nichols J., Sobel M., Lee A., Somkuti S., Hirshberg S., Budinetz T., Barmat L., Palermo G., Rosenwaks Z., Bar-Chama N., Bodie J. (2020). Multicentric, prospective observational data show sperm capacitation predicts male fertility, and cohort comparison reveals a high prevalence of impaired capacitation in men questioning their fertility. Reprod. Biomed. Online.

[15] Luque G.M., Dalotto-Moreno T., Martin-Hidalgo D., Ritagliati C., Puga Molina L.C., Romarowski A., Balestrini P.A., Schiavi-Ehrenhaus L.J., Gilio N., Krapf D., Visconti P.E., Buffone M.G. (2018). Only a subpopulation of mouse sperm displays a rapid increase in intracellular calcium during capacitation. J. Cell Physiol..

[16] Travis A.J., Kopf G.S. (2002). The role of cholesterol efflux in regulating the fertilization potential of mammalian spermatozoa. J. Clin. Invest..

[17] Cohen R., Mukai C., Travis A.J. (2016). Lipid regulation of acrosome exocytosis. Adv. Anat. Embryol. Cell Biol..

[18] Puga Molina L.C., Luque G.M., Balestrini P.A., Marin-Briggiler C.I., Romarowski A., Buffone M.G. (2018). Molecular basis of human sperm capacitation. Front. Cell Dev. Biol..

[19] Holt W.V., North R.D. (1984). Partially irreversible cold-induced lipid phase transitions in mammalian sperm plasma membrane domains: Freeze-fracture study. J. Exp. Zool.

[20] Wolf D.E., Maynard V.M., McKinnon C.A., Melchior D.L. (1990). Lipid domains in the ram sperm plasma membrane demonstrated by differential scanning calorimetry. Proc. Natl. Acad. Sci. U. S. A..

[21] Asano A., Nelson-Harrington J.L., Travis A.J. (2013). Membrane rafts regulate phospholipase B activation in murine sperm. Commun. Integr. Biol..

[22] Asano A., Nelson-Harrington J.L., Travis A.J. (2013). Phospholipase B is activated in response to sterol removal and stimulates acrosome exocytosis in murine sperm. J. Biol. Chem..

[23] Cohen R., Buttke D.E., Asano A., Mukai C., Nelson J.L., Ren D., Miller R.J., Cohen-Kutner M., Atlas D., Travis A.J. (2014). Lipid modulation of calcium flux through CaV2.3 regulates acrosome exocytosis and fertilization. Dev. Cell.

[24] Lishko P.V., Kirichok Y. (2010). The role of Hv1 and CatSper channels in sperm activation. J. Physiol..

[25] Wang D., King S.M., Quill T.A., Doolittle L.K., Garbers D.L. (2003). A new sperm-specific Na+/H+ exchanger required for sperm motility and fertility. Nat. Cell Biol..

[26] Santi C.M., Martinez-Lopez P., de la Vega-Beltran J.L., Butler A., Alisio A., Darszon A., Salkoff L. (2010). The SLO3 sperm-specific potassium channel plays a vital role in male fertility. FEBS Lett..

[27] De La Vega-Beltran J.L., Sanchez-Cardenas C., Krapf D., Hernandez-Gonzalez E.O., Wertheimer E., Trevino C.L., Visconti P.E., Darszon A. (2012). Mouse sperm membrane potential hyperpolarization is necessary and sufficient to prepare sperm for the acrosome reaction. J. Biol. Chem..

[28] Chavez J.C., De la Vega-Beltran J.L., Jose O., Torres P., Nishigaki T., Trevino C.L., Darszon A. (2018). Acrosomal alkalization triggers Ca(2+) release and acrosome reaction in mammalian spermatozoa. J. Cell Physiol..

[29] de Lamirande E., Harakat A., Gagnon C. (1998). Human sperm capacitation induced by biological fluids and progesterone, but not by NADH or NADPH, is associated with the production of superoxide anion. J. Androl..

[30] Uhler M.L., Leung A., Chan S.Y., Wang C. (1992). Direct effects of progesterone and antiprogesterone on human sperm hyperactivated motility and acrosome reaction. Fertil. Steril..

[31] Harper C.V., Barratt C.L., Publicover S.J. (2004). Stimulation of human spermatozoa with progesterone gradients to simulate approach to the oocyte. Induction of [Ca(2+)](i) oscillations and cyclical transitions in flagellar beating. J. Biol. Chem..

[32] Teves M.E., Barbano F., Guidobaldi H.A., Sanchez R., Miska W., Giojalas L.C. (2006). Progesterone at the picomolar range is a chemoattractant for mammalian spermatozoa. Fertil. Steril..

[33] Calogero A.E., Burrello N., Barone N., Palermo I., Grasso U., D'Agata R. (2000). Effects of progesterone on sperm function: Mechanisms of action. Hum. Reprod..

[34] Francavilla F., Romano R., Santucci R., Macerola B., Ruvolo G., Francavilla S. (2002). Effect of human sperm exposure to progesterone on sperm-oocyte fusion and sperm-zona pellucida binding under various experimental conditions. Int. J. Androl..

[35] Sagare-Patil V., Galvankar M., Satiya M., Bhandari B., Gupta S.K., Modi D. (2012). Differential concentration and time dependent effects of progesterone on kinase activity, hyperactivation and acrosome reaction in human spermatozoa. Int. J. Androl..

[36] Kirkman-Brown J.C., Bray C., Stewart P.M., Barratt C.L., Publicover S.J. (2000). Biphasic elevation of [Ca(2+)](i) in individual human spermatozoa exposed to progesterone. Dev. Biol..

[37] Navarro B., Kirichok Y., Chung J.J., Clapham D.E. (2008). Ion channels that control fertility in mammalian spermatozoa. Int. J. Dev. Biol..

[38] Sun X.H., Zhu Y.Y., Wang L., Liu H.L., Ling Y., Li Z.L., Sun L.B. (2017). The Catsper channel and its roles in male fertility: A systematic review. Reprod. Biol. Endocrinol..

[39] Nakanishi T., Ikawa M., Yamada S., Parvinen M., Baba T., Nishimune Y., Okabe M. (1999). Real-time observation of acrosomal dispersal from mouse sperm using GFP as a marker protein. FEBS Lett..

[40] Hasuwa H., Muro Y., Ikawa M., Kato N., Tsujimoto Y., Okabe M. (2010). Transgenic mouse sperm that have green acrosome and red mitochondria allow visualization of sperm and their acrosome reaction *in vivo*. Exp. Anim..

[41] Herrick S.B., Schweissinger D.L., Kim S.W., Bayan K.R., Mann S., Cardullo R.A. (2005). The acrosomal vesicle of mouse sperm is a calcium store. J. Cell. Physiol..

[42] Henderson M.J., Baldwin H.A., Werley C.A., Boccardo S., Whitaker L.R., Yan X., Holt G.T., Schreiter E.R., Looger L.L., Cohen A.E., Kim D.S., Harvey B.K. (2015). A low affinity GCaMP3 variant (GCaMPer) for imaging the endoplasmic reticulum calcium store. PLoS One.

[43] Shui B., Lee J.C., Reining S., Lee F.K., Kotlikoff M.I. (2014). Optogenetic sensors and effectors: CHROMus-the cornell heart lung Blood institute resource for optogenetic mouse signaling. Front. Physiol..

[44] Amatore C., Arbault S., Bonifas I., Guille M. (2009). Quantitative investigations of amperometric spike feet suggest different controlling factors of the fusion pore in exocytosis at chromaffin cells. Biophys. Chem..

[45] Ikawa M., Yamada S., Nakanishi T., Okabe M. (1998). 'Green mice' and their potential usage in biological research. FEBS Lett..

[46] Pitelka D.R., Taggart B.N., Hamamoto S.T. (1983). Effects of extracellular calcium depletion on membrane topography and occluding junctions of mammary epithelial cells in culture. J. Cell Biol..

[47] Abi Nahed R., Martinez G., Escoffier J., Yassine S., Karaouzene T., Hograindleur J.P., Turk J., Kokotos G., Ray P.F., Bottari S., Lambeau G., Hennebicq S., Arnoult C. (2016). Progesterone-induced acrosome exocytosis requires sequential involvement of calcium-independent phospholipase A2beta (iPLA2beta) and group X secreted phospholipase A2 (sPLA2). J. Biol. Chem..

[48] Hino T., Oda K., Nakamura K., Tateno H., Toyoda Y., Yokoyama M. (2011). Accelerated modification of the zona pellucida is the primary cause of decreased fertilizability of oocytes in the 129 inbred mouse strain. Zygote.

[49] Costello S., Michelangeli F., Nash K., Lefievre L., Morris J., Machado-Oliveira G., Barratt C., Kirkman-Brown J., Publicover S. (2009). Ca2+-stores in sperm: Their identities and functions. Reproduction.

[50] Walensky L.D., Snyder S.H. (1995). Inositol 1,4,5-trisphosphate receptors selectively localized to the acrosomes of mammalian sperm. J. Cell Biol..

[51] Arndt L., Castonguay J., Arlt E., Meyer D., Hassan S., Borth H., Zierler S., Wennemuth G., Breit A., Biel M., Wahl-Schott C., Gudermann T., Klugbauer N., Boekhoff I. (2014). NAADP and the two-pore channel protein 1 participate in the acrosome reaction in mammalian spermatozoa. Mol. Biol. Cell.

[52] Vasudevan S.R., Lewis A.M., Chan J.W., Machin C.L., Sinha D., Galione A., Churchill G.C. (2010). The calcium-mobilizing messenger nicotinic acid adenine dinucleotide phosphate participates in sperm activation by mediating the acrosome reaction. J. Biol. Chem..

[53] Lawson C., Dorval V., Goupil S., Leclerc P. (2007). Identification and localisation of SERCA 2 isoforms in mammalian sperm. Mol. Hum. Reprod..

[54] Li S., Hao B., Lu Y., Yu P., Lee H.C., Yue J. (2012). Intracellular alkalinization induces cytosolic Ca2+ increases by inhibiting sarco/endoplasmic reticulum Ca2+-ATPase (SERCA). PLoS One.

[55] Williams K.M., Ford W.C. (2003). Effects of Ca-ATPase inhibitors on the intracellular calcium activity and motility of human spermatozoa. Int. J. Androl..

[56] Wennemuth G., Babcock D.F., Hille B. (2003). Calcium clearance mechanisms of mouse sperm. J. Gen. Physiol..

[57] Blackmore P.F. (1999). Extragenomic actions of progesterone in human sperm and progesterone metabolites in human platelets. Steroids.

[58] O'Toole C.M., Arnoult C., Darszon A., Steinhardt R.A., Florman H.M. (2000). Ca(2+) entry through store-operated channels in mouse sperm is initiated by egg ZP3 and drives the acrosome reaction. Mol. Biol. Cell.

[59] Correia J., Michelangeli F., Publicover S. (2015). Regulation and roles of Ca2+ stores in human sperm. Reproduction.

[60] Darszon A., Sanchez-Cardenas C., Orta G., Sanchez-Tusie A.A., Beltran C., Lopez-Gonzalez I., Granados-Gonzalez G., Trevino C.L. (2012). Are TRP channels involved in sperm development and function?. Cell Tissue Res..

[61] Morris J., Jones S., Howl J., Lukanowska M., Lefievre L., Publicover S. (2015). Cell-penetrating peptides, targeting the regulation of store-operated channels, slow decay of the progesterone-induced [Ca2+]i signal in human sperm. Mol. Hum. Reprod..

[62] Nishigaki T., Jose O., Gonzalez-Cota A.L., Romero F., Trevino C.L., Darszon A. (2014). Intracellular pH in sperm physiology. Biochem. Biophys. Res. Commun..

[63] Nakanishi T., Ikawa M., Yamada S., Toshimori K., Okabe M. (2001). Alkalinization of acrosome measured by GFP as a pH indicator and its relation to sperm capacitation. Dev. Biol..

[64] Helassa N., Zhang X.H., Conte I., Scaringi J., Esposito E., Bradley J., Carter T., Ogden D., Morad M., Torok K. (2015). Fast-response calmodulin-based fluorescent indicators reveal rapid intracellular calcium dynamics. Sci. Rep..

[65] Naaby-Hansen S., Wolkowicz M.J., Klotz K., Bush L.A., Westbrook V.A., Shibahara H., Shetty J., Coonrod S.A., Reddi P.P., Shannon J., Kinter M., Sherman N.E., Fox J., Flickinger C.J., Herr J.C. (2001). Co-localization of the inositol 1,4,5-trisphosphate receptor and calreticulin in the equatorial segment and in membrane bounded vesicles in the cytoplasmic droplet of human spermatozoa. Mol. Hum. Reprod..

[66] Mills T.T., Huang J., Feigenson G.W., Nagle J.F. (2009). Effects of cholesterol and unsaturated DOPC lipid on chain packing of saturated gel-phase DPPC bilayers. Gen. Physiol. Biophys..

[67] Travis A.J., Tutuncu L., Jorgez C.J., Ord T.S., Jones B.H., Kopf G.S., Williams C.J. (2004). Requirements for glucose beyond sperm capacitation during *in vitro* fertilization in the mouse. Biol. Reprod..

[68] Travis A.J., Jorgez C.J., Merdiushev T., Jones B.H., Dess D.M., Diaz-Cueto L., Storey B.T., Kopf G.S., Moss S.B. (2001). Functional relationships between capacitation-dependent cell signaling and compartmentalized metabolic pathways in murine spermatozoa. J. Biol. Chem..

