# Supplementary material for: A genetically targeted sensor reveals spatial and temporal dynamics of acrosomal calcium and sperm acrosome exocytosis
Source: J Biol Chem. 2022 Mar 27;298(5):101868. doi: 10.1016/j.jbc.2022.101868 (PMC9046242; doi:10.1016/j.jbc.2022.101868)

**A Genetically-targeted sensor reveals spatial and temporal dynamics of acrosomal calcium and sperm acrosome exocytosis**

**SUPPORTING INFORMATION**

**Supporting Figure 1.**

**Coomassie AE assay**. A) Sperm from 3 mice (>180 sperm counted for each condition) were collected and incubated in 37°C in the presence 10mM Ca^2+^ along with either G_M1_ (125uM), or CD (3mM) or both. Following a 30 minute incubation, cells were stained with coomassie and counted for AE. B) as in A, but incubation was for 90 minutes.


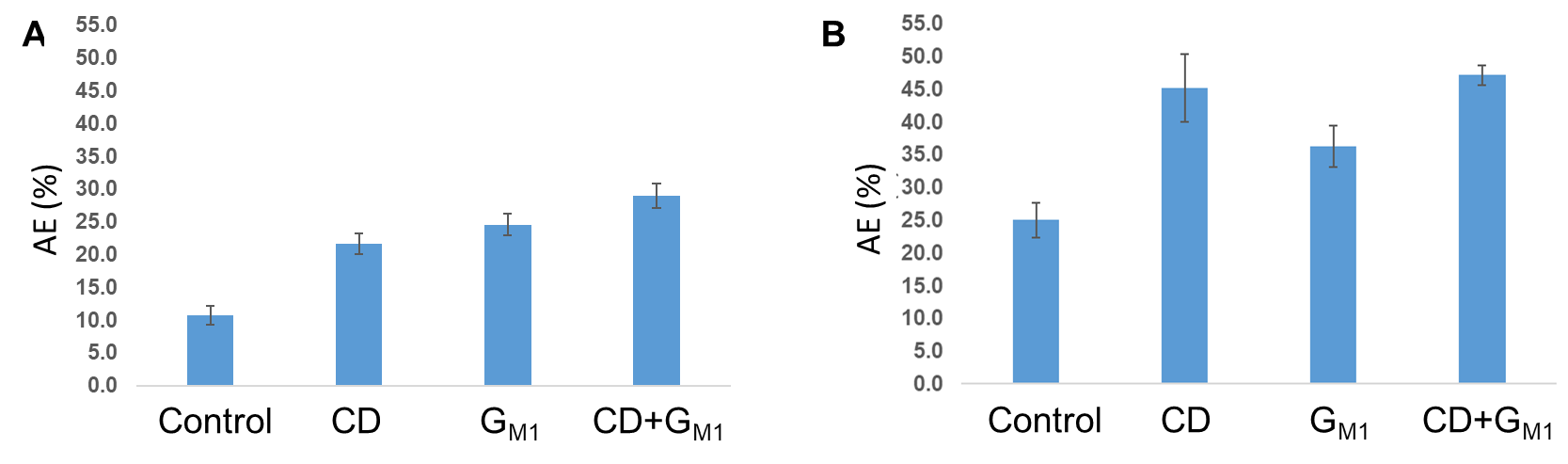


**Supporting Figure 2.**

**Statistical analysis (c^2^) for Figure 3B. A)** Chi-square test was applied to ACR and MF events in response to the different stimulations as indicated in Figure 3B. **c^2^** values (top chart) and p values (bottom chart) are presented for ACR (A) and MF (B).


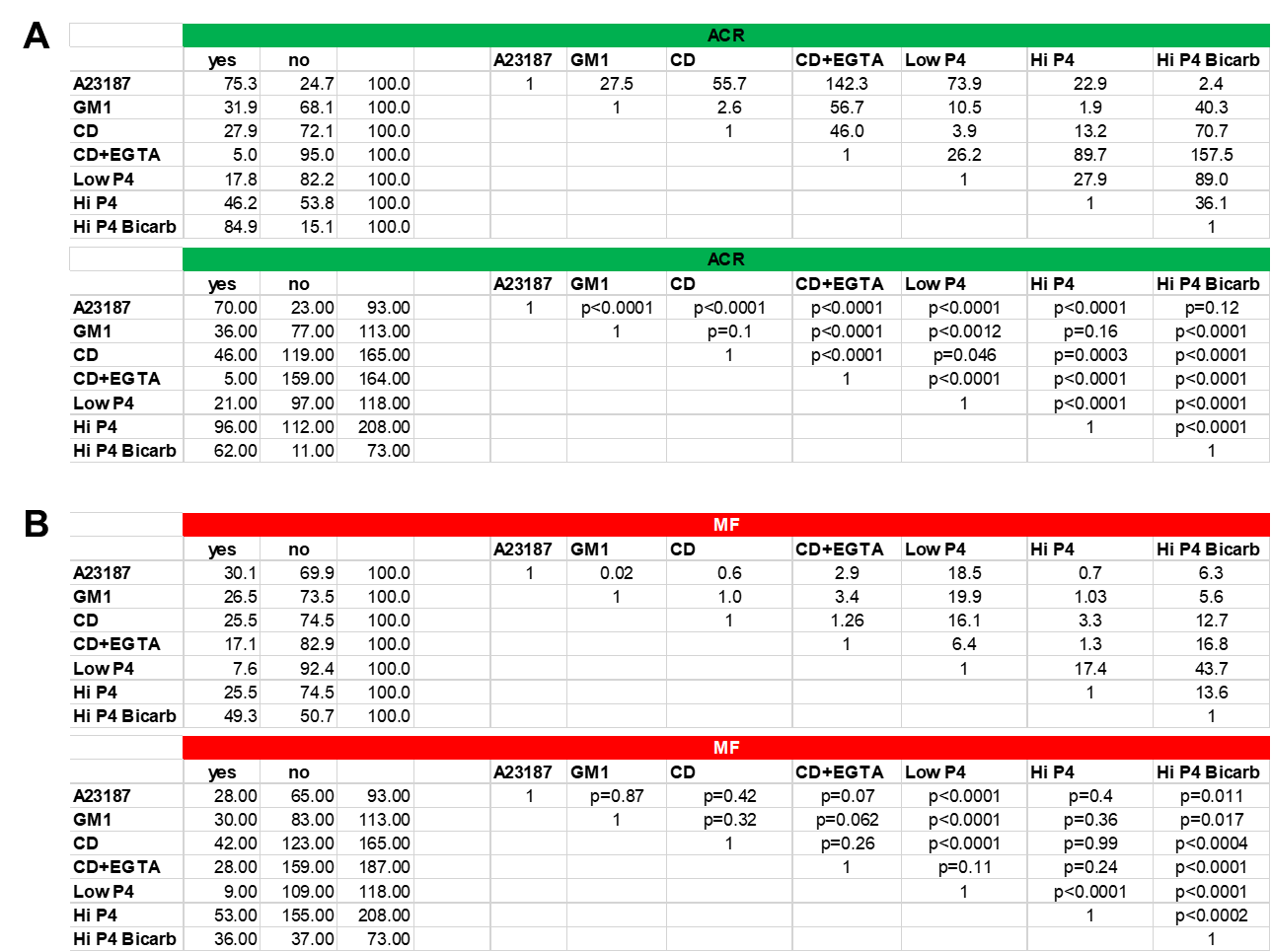


**Supporting Figure 3.**

**Chi^2^ analysis of Figure 5A. c^2^** analysis confirms significant difference between the paired data sets (p<<0.05).

**Supporting Figure 4.**

**Chi^2^ analysis of Figure 5B. c^2^** analysis confirms significant difference between the paired data sets (p<<0.05), except for the case of Hi-P4/CD where high correlation was found (p=0.39).

**Supporting Figure 5.**

**Chi^2^ analysis of Figure 5C. c^2^** analysis confirms significant difference between the paired data sets (p<<0.05), except for the case of GM1+bicarb/Hi-P4 where high correlation was found (p=0.69).

**Supporting Figure 6.**

**t-test analysis of panels in Figure 6.** t-test analysis was applied between every 2 conditions for (separately for each of the panels). Numbers indicate the p values for each test.


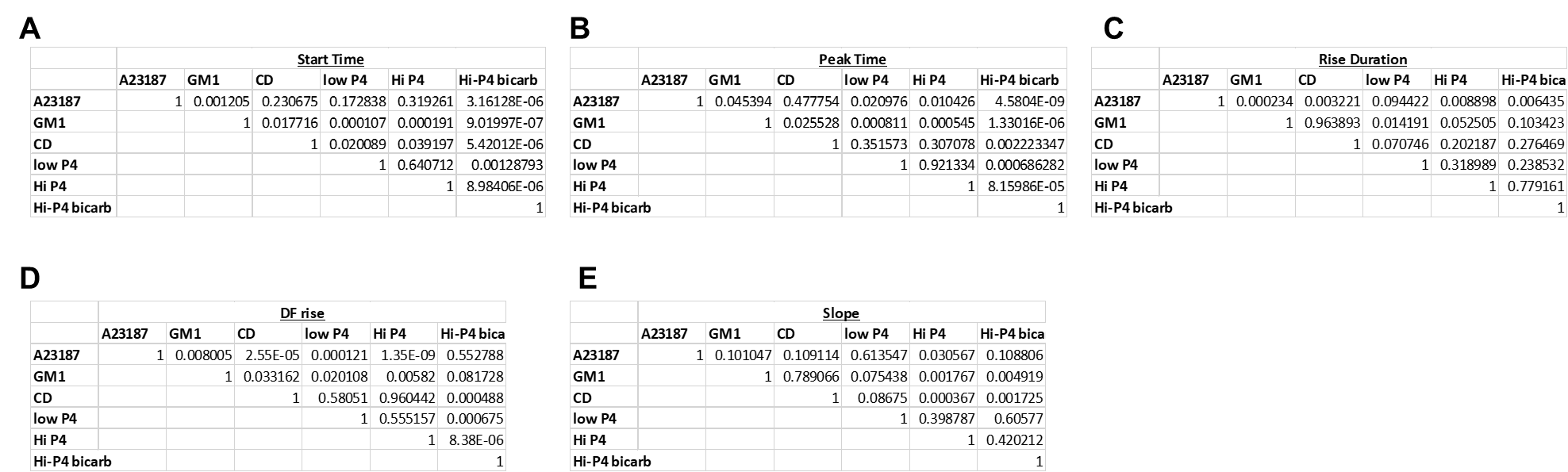


**Supporting Figure 7.**

**t-test analysis of panels in Figure 8.** t-test analysis was applied between every 2 conditions for (separately for each of the panels). Numbers indicate the p values for each test.


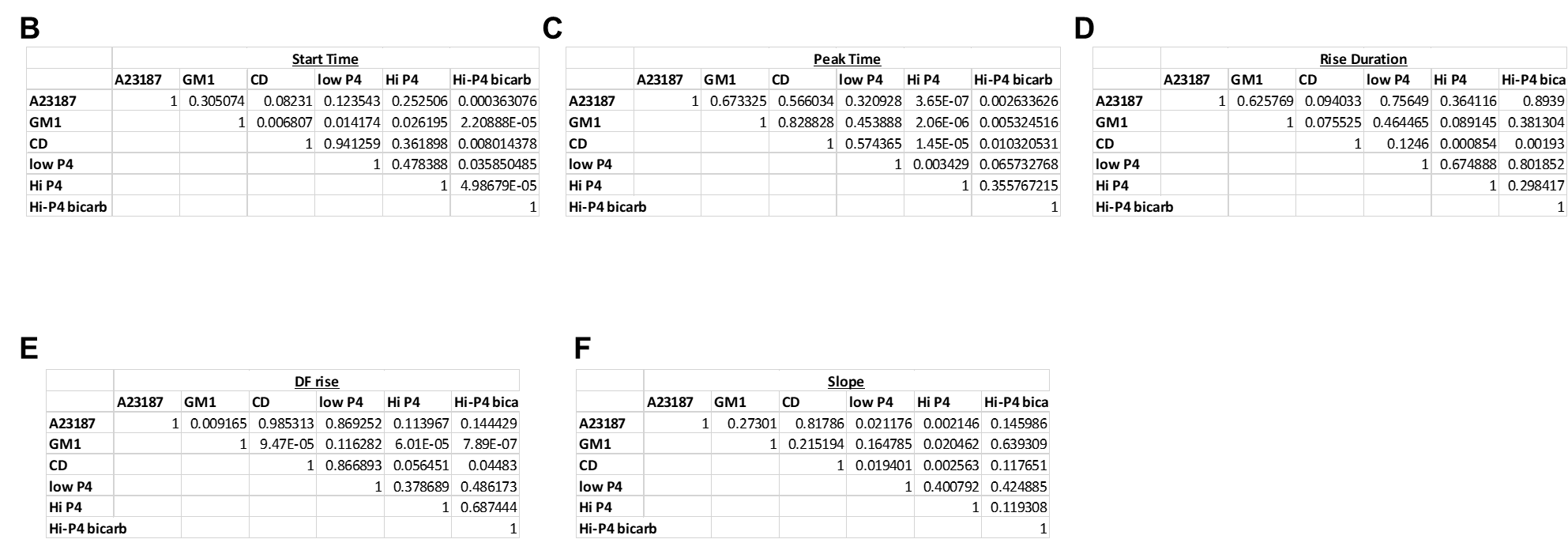

Supplement: Supporting Figures S1–S7 [file mmc1.docx]
